# Coevolution of actions, personal norms and beliefs about others in social dilemmas

**DOI:** 10.1017/ehs.2021.40

**Published:** 2021-08-19

**Authors:** Sergey Gavrilets

**Affiliations:** Department of Ecology and Evolutionary Biology, Department of Mathematics, National Institute for Mathematical and Biological Synthesis, Center for the Dynamics of Social Complexity, University of Tennessee, Knoxville, TN 37996 USA

**Keywords:** Cooperation, conflict, cultural evolution, social evolution, mathematical models

## Abstract

Human decision-making is affected by a diversity of factors including material cost–benefit considerations, normative and cultural influences, learning and conformity with peers and external authorities (e.g. cultural, religious, political, organisational). Also important are dynamically changing personal perceptions of the situation and beliefs about actions and expectations of others as well as psychological phenomena such as cognitive dissonance and social projection. To better understand these processes, I develop a unifying modelling framework describing the joint dynamics of actions and attitudes of individuals and their beliefs about the actions and attitudes of their groupmates. I consider which norms get internalised and which factors control beliefs about others. I predict that the long-term average characteristics of groups are largely determined by a balance between material payoffs and the values promoted by the external authority. Variation around these averages largely reflects variation in individual costs and benefits mediated by individual psychological characteristics. The efforts of an external authority to change the group behaviour in a certain direction can, counter-intuitively, have an opposite effect on individual behaviour. I consider how various factors can affect differences between groups and societies in the tightness/looseness of their social norms. I show that the most important factors are social heterogeneity, societal threat, effects of authority, cultural variation in the degree of collectivism/individualism, the population size and the subsistence style. My results can be useful for achieving a better understanding of human social behaviour and historical and current social processes, and in developing more efficient policies aiming to modify social behaviour.

## Graphical abstract

**Figure:**
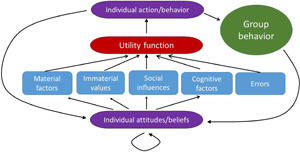

**Social media summary:** A unifying modelling framework predicts the effects of material, social and cognitive forces on human behaviour and beliefs.

## Introduction

Human groups at various scales of social organisation repeatedly face situations where engaging in an individually costly collective action or refraining from an individually beneficial behaviour can help bring larger benefits or avoid certain disastrous outcomes. Examples range from cooperating in hunting or agricultural production in small-scale societies to mobilising against social injustice to modifying the collective behaviour of the population to stop a pandemic or decrease global warming. Such situations commonly lead to social dilemmas when individual and group interests come into a conflict. In the scientific literature, they come under various names including the collective action problem (Olson, 1965; Pecorino, 2015), the tragedy of the commons (Hardin, 1968, Ostrom, 2000), social traps (Platt, 1973), the many-person Prisoner's Dilemma (Schelling, 1978; Molander, 1992) and the collective risk dilemma (Milinski et al., 2008).

Human decision-making in social dilemmas is affected by a diversity of factors including genetically informed biological instincts, material cost–benefit considerations, normative and cultural influences, and conformity with peers or external authorities (e.g. cultural, religious, political, organisational). Human actions also depend on their personal perception of the situation and on beliefs about the actions and expectations of their peers (R. L. Cialdini et al., 1990; Troyer and Younts, 1997; Bicchieri, 2006). The beliefs and expectations can change as a result of learning and other psychological processes. For example, cognitive dissonance (i.e. a feeling of mental discomfort experienced when the person's attitudes, beliefs or behaviours conflict) can cause changes in behaviours but also in attitudes or beliefs (Festinger, 1957). To predict the intentions and beliefs of others, people may use the ‘theory of mind’ (Premack and Wodruff, 1979; Apperly, 2010) and social projection, which is the tendency to assume that others are similar to oneself (Krueger, 2007). Therefore changing personal attitudes can also change predictions about others.

Owing to this complexity, modelling human behaviour is notoriously difficult. Nevertheless several approaches successfully capturing certain aspects of human decision-making have been developed. These include classical (Fudenberg and Tirole, 1992), evolutionary (Sandholm, 2010), mean-field (Tembine, 2017) and quantum (Piotrowski and Sladkowski, 2003; Siopsis et al., 2018) game theories focusing on the effects of material payoffs, social influence models focusing on the dynamics of consensus formation (or fragmentation) in social networks as a result of social learning and imitation (DeGroot, 1974; Watts, 2002; Friedkin et al., 2016; Redner, 2019; Galesic and Stein, 2019; Zino et al., 2020; Kashima et al., 2021), models of strategic deliberation (Golman et al., 2020), models of normative behaviour (Azar, 2004; S. Gavrilets and Richerson, 2017; S. Gavrilets, 2020) and models of foresight (Perry et al., 2018; Perry and Gavrilets, 2020). Each of these approaches concentrates on specific forces shaping human behaviour and beliefs while neglecting many other important factors.

Here I will build on this earlier work to develop a novel theoretical approach explicitly integrating multiple material, cognitive, emotional and social forces shaping human behaviour. I posit that individuals are motivated by both material factors and values and norms, that their actions are driven by their interpretation of what they observe and that their interpretations and beliefs change dynamically as social interactions unfold. In my theoretical approach, the individual's actions and beliefs are influenced by their social environment as well as by certain internal psychological processes. Mathematically, these assumptions are implemented by adding several additional components besides material payoffs to the utility function and by writing down coupled equations specifying the dynamics of attitudes and beliefs about others.

My approach aims to shed theoretical light on a number of important questions: how can individuals find the right action when facing social dilemmas? Which factors (material, social, psychological) are most important in their decisions? What happens to their preferences, beliefs and behaviours as social interactions unfold dynamically? Which social norms get internalised? Which factors control individual beliefs about others? How different are the effects of peer influences from those of an external authority? What are the effects of between-individual differences in physical, social and psychological characteristics on group behaviour? How robust are game-theoretic predictions on short and long time scales in the presence of non-material influences and belief dynamics? Which psychological forces are most powerful? What are the cultural effects on individual and group behaviour? How is the tightness or looseness of social norms related to various environmental, social and psychological forces? My approach also offers a way to measure and compare the relative strengths of different factors affecting individual actions and beliefs.

**Figure 1.**
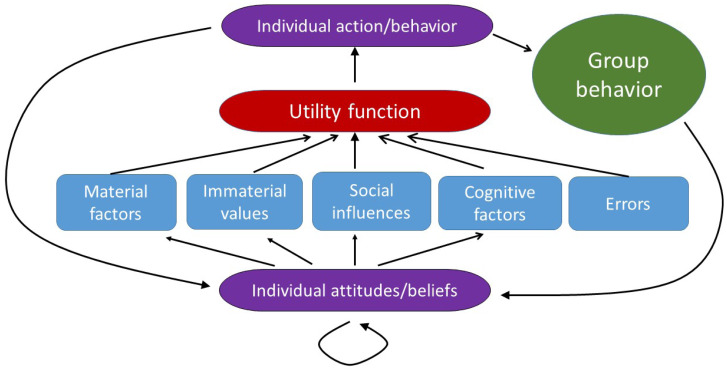
Model structure. The model integrates material factors, nonmaterial values, social influences (both by peers and an external authority), cognitive factors and errors (the blue boxes) into a general utility function (the red shape) which individuals attempt to maximise when making decisions (the top violet shape). Individual behavior is a part of group behavior (the green shape). Individual actions taken and observed group behavior as well as previous attitudes and beliefs feed back into updated individual beliefs and attitudes (bottom violet shape). In my approach, the strength of various factors, as perceived by individuals, will vary between them depending on the information available as well as on the individuals’ attitudes and beliefs. My approach allows for attitudes and beliefs to (rapidly) change in time as a consequence of different actions taken by individuals and the groups they belong to, the information they receive and the emotions they experience.

My starting point is what is known in social psychology as the ‘Thomas theorem’, which states that ‘If men define situations as real, they are real in their consequences’ (Thomas, 1928). In other words, our actions often depend on our interpretation of a situation rather than on its objective reality. In my models, I will capture this ‘theorem’ by postulating that individual decisions in social situations are based on individual beliefs about the current situation as well as beliefs about others and their beliefs. Individuals will revise their actions, attitudes and beliefs according to not only the information they receive but also some psychological processes governing their thinking and emotions (Wood, 2000; Albarracin and Shavitt, 2017). The general structure of my model is illustrated in Figure 1.

Below after introducing my approach and describing main results, I illustrate them by considering different types of social interactions including those stylised by Coordination, Public Goods, Tragedy of the Commons, Common Pool Resource, continuous Prisoner's Dilemma, Dictator and ‘Us vs. nature’ games. At the end, I discuss the implications of my results for empirical and theoretical research on the behaviour and beliefs of individuals and groups.

## Model

I consider a group of people repeatedly engaged in a particular type of social interaction. For example, individuals can contribute efforts to a joint production or maintenance of a public good (e.g. an irrigation canal) or harvest from a common pool of resources (e.g. fishing from a pond). Individuals care about their own material costs and benefits. They do not like to be disapproved by peers (or an external authority) but they also prefer to do what they personally think is appropriate. Individuals are bounded rational (Simon, 1957). They observe (and learn from) the actions of others and make inferences about others’ attitudes (preferences) and beliefs but they do not know them exactly. How can they find the right action? What happens to their preferences, beliefs and behaviours as social interactions dynamically unfold?

I will treat time as discrete. Let a continuous variable *x* specify an action chosen by a focal individual. Each individual is characterised by an attitude *y* which gives his personal belief about the most appropriate action in a given social situation. Each individual also has a belief (an expectation) 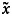 about the average action of peers as well as a second order belief 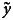 about the average attitude of their peers. Experiments show that people represent the preferences and beliefs of others separately from their own (Hedden and Zhang, 2002; Goodie et al., 2012; Jamali et al., 2021). In the social psychology literature, variables 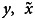, and 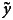 would be called a personal norm (or value), an empirical expectation and a normative expectation, respectively (Bicchieri, 2006; Bicchieri et al., 2020; Szekely et al., 2021). Below I will use the terms ‘attitude’ and ‘personal norm’ interchangeably. Individuals are also subject to influence by an external authority promoting a particular action *G*. I assume that 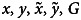 are non-negative. I note that recent work directly measures these variables in behavioural experiments (Szekely et al., 2021; d’Adda et al., 2020; Andreozzi et al., 2020; Basić and Verrina, 2020; Kölle and Quercia, 2021). Individuals form their beliefs about others on the basis of the actions they observe and some cognitive and psychological processes (which I discuss below).

### Utility function

I postulate that each individual chooses (via myopic best response) an action *x* in an attempt to maximise the (subjective) utility function *u*. I write it as a sum of several terms:1
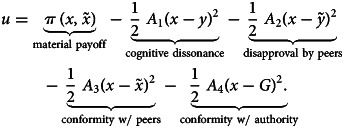
The first term in equation (1) specifies a material payoff to a focal individual performing action *x* under the expectation that his peers’ average action is 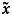. The second term in equation (1) captures the psychic costs owing to cognitive dissonance (Festinger, 1957) incurred when the action *x* chosen deviates from the personal norm *y*. The third term captures the expected psychic costs of disapproval (or material costs of punishment) by others who are expected to have expectation 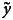 regarding the behaviour of the focal individual (R. L. Cialdini et al., 1990; Bicchieri, 2006). The fourth term in equation (1) captures the psychic costs of non-conformity with the expected actions of others (R. B. Cialdini and Goldstein, 2004; Song et al., 2012). For example, the fact that peers choose a particular action may indicate that this action is most beneficial. So acting differently may cause additional psychic costs not related to disapproval or punishment by peers (captured by the third term). The last term in equation (1) captures the expected costs of material punishment or psychic costs of disapproval by the external authority promoting an action at a ‘standard’ level *G* which I will treat as a constant (French and Raven, 1959; R. B. Cialdini and Goldstein, 2004). Some studies show stable variation between people in following the ‘rules’ (Kimbrough and Vostroknutov, 2016, 2018).

I assume that parameters *A*_1_, *A*_2_, *A*_3_ and *A*_4_ are non-negative individual-specific constants. This assumption aims to capture the fact that people differ in their personalities, cultural background and other characteristics affecting their emotions, feelings, psychology and behaviour. Parameters *A*_2_ and *A*_3_ may depend on the group size, so that individuals whose actions deviate from the expected behaviour or beliefs of others suffer bigger costs in larger groups. Parameter *A*_4_ may depend on the degree of legitimacy of the external authority and on individual self-identification.

My approach is particularly simple when the function 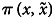 specifying the material payoff is a linear, quasi-linear or a quadratic function of *x* and 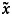. For such cases, the first derivative of 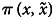 (i.e. marginal payoff) with respect to *x* is a linear function of *x* and 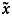, which I will write as2
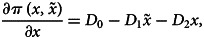
where *D*_0_, *D*_1_ and *D*_2_ are constant individual-specific parameters. For example, individuals may differ in their strengths, valuation (or shares received) of the collectively produced goods, costs or availability of information regarding the material consequences of the game. [For simplicity of notation, for now I do not use explicitly any indices in the equations to specify the individual. This will change later when I discuss specific social situations and games.]

Below I will use a composite parameter of the material payoff function3
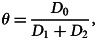
which can be interpreted as the best response action for a focal individual who believes that the average action of his social partners will always match his own action (i.e. 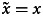). In several games to be considered below, *θ* can also be viewed as a measure of the material benefit-to-cost ratio; in some games *θ* is the Nash equilibrium for the individual effort. As I show below, the distribution of *θ* in the society strongly affects the long-term dynamics of the model. When I use agent-based simulations, I will also allow for errors in decision-making.

### Best response action

The action *x* maximising the utility function *u* of the focal individual can be found by computing the derivative ∂*u*/∂*x*. Since *u* is a quadratic function, the best response action given an attitude *y* and beliefs 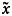 and 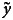 can be found in a straightforward way. I will write it as4
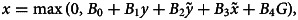
where *B*_0_, …, *B*_4_ are re-scaled individual-specific parameters measuring the effects of material and non-material forces on individual actions (see the Supporting Information). I assume that all individuals in the group take their own best response actions simultaneously.

### The dynamics of attitudes and beliefs

After taking their own action and observing the actions of their groupmates, each individual revises their attitudes and beliefs. To capture these changes, I adapt an approach standard in social influence models describing the dynamics of publicly expressed opinions. Specifically I postulate that attitudes and beliefs of a focal individual change according to a system of linear recurrence equations:5*a*
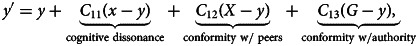
5*b*
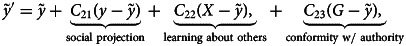
5*c*
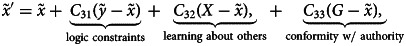
where the prime means that the next time step, *X* is the average action of groupmates as observed by the focal individual (so that different individuals are characterised by different *X*) and *C*_*ij*_ represents non-negative individual-specific constant coefficients. Here the ‘cognitive dissonance’ term acts to reduce the mismatch of the ego's actions and their beliefs about themselves. The ‘social projection’ term captures the ego's belief that others are probably similar to themselves (Premack and Wodruff, 1979; Krueger, 2007). The ‘logic constraints’ term reduces a mismatch between the ego's beliefs about actions and beliefs of others (cf. Friedkin et al. 2016). The ‘conformity w/ peers’ and two ‘learning about others’ terms move the corresponding attitude and beliefs closer to the observed average behaviour *X* among peers (Kashima et al., 2015). The ‘conformity w/ authority’ terms move the corresponding attitudes and beliefs closer to the promoted ‘standard’ *G*. Note that cognitive dissonance makes individuals choose action *x* closer to their attitude *y* (as implied by equation 1) and simultaneously changes their attitude *y* to justify the action previously chosen (as described by the first term in equation (5a); cf. Rabin 1994). The authority effectively changes the utility function (1) and simultaneously affects attitudes and beliefs (equations 5), which then feed back into the utility function and behaviour. For a group of *n* individuals I thus end up with 3*n* recurrence equations of type (5) which are coupled via terms *X* which are the observed average actions of groupmates. Below in deriving analytical approximations I will assume that *n* is sufficiently large that individual values *X* are approximately the same (and equal to the actual average action of the group).

Below I will use normalised parameters
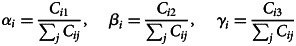
with *α*_*i*_ + *β*_*i*_ + *γ*_*i*_ = 1 for all *i*. Parameter *α*_*i*_ characterises the relative strengths of cognitive factors (i.e. related to the cognitive dissonance, the social projection, and the logic constraint, respectively). Parameters *β*_*i*_ and *γ*_*i*_ characterise the relative strengths of two types of social factors: learning from/about peers and complying with external influences, respectively. All of these coefficients are individual specific; they may depend on individual psychology, cultural and education background, etc. They may also depend on social and cultural factors acting in the group. For example, increased efforts to promote certain ideas by an authority may translate in increased values of parameters *γ*_*i*_ while strongly conformist or collectivistic communities may be characterised by higher values of parameters *β*_*i*_. Parameters *B*_4_ and *γ*_*i*_ can depend on trust in the authority and its legitimacy. Intuitively, cognitive factors work to align individual actions, attitude and beliefs, learning from/about peers works to align those between individuals, while external influence works to shift them towards a promoted standard.

Before proceeding further it is instructive to compare my approach with already existing models. First, classical, evolutionary and mean-field game-theoretic models focus exclusively on the material payoff component *π* (*x*) of the utility model disregarding all other terms (Fudenberg and Tirole, 1992; Sandholm, 2010; Tembine, 2017; Gomes and Saúde, 2014). Note also that in contrast to standard evolutionary game theory models where individuals myopically choose the best responses to the previous action of their mates which they know exactly, in my approach they best respond to their expectation 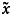 of the action of their group-mates in this round. Some game-theoretic models add a normative component to the utility function but treat personal norms *y* as constant (Azar, 2004; S. Gavrilets and Richerson, 2017; S. Gavrilets, 2020). Relatively few existing models consider the joint dynamics of actions (*x*) and personal norms (*y*). For example in Rabin (1994), Kuran and Sandholm (2008) and Calabuig et al. (2018), utility functions include material payoffs *π* (*x*) as well cognitive dissonance and conformity with peers terms. Kuran and Sandholm (2008) and Calabuig et al. (2018) describe the dynamics of personal norms *y* allowing for the effects of cognitive dissonance and conformity with peers. Bisin and Verdier (2001) and Cheung and Wu (2018) consider the inter-generation evolution of preferences. However all of these papers assume that individuals know exactly the personal norms *y* of their peers which in general is not realistic. There is also a very large number of social influence models (DeGroot, 1974; Watts, 2002; Friedkin et al., 2016; Redner, 2019; Galesic and Stein, 2019; Kashima et al., 2021; Centola et al., 2005) which consider the dynamics of personal attitudes and opinions *y* as a result of the exchange of opinions between group members (using linear equations related to the second and third terms in equation 5a). The linear equations describing the changes in attitudes and beliefs are also related to those used in cognitive neuroscience (Olsson et al., 2020). Focusing on dyadic interactions, Golman et al. (2020) model how individuals update their values of *y* and 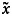 on the basis of payoffs received. Y. N. Gavrilets (2003) considered similar models but with the addition of an external influence (described by a term analogous to the last term in equation 5a). Models of social influence neglect material factors, and explicitly assume that players know exactly the opinions of their peers. None of all these models consider second-order beliefs of individuals captured by variables 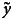 and 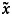.

I note that the model's structure reflects the facts that human behaviour and beliefs are complex phenomena and that real people differ in their psychology and behaviour. As I show below, in spite of its apparent complexity, the model's behaviour is quite tractable, its parameters combine into a small number of effective measures controlling the equilibria and individual parameters can be estimated using behavioural economics methods or surveys.

## Results

### Long-term behaviour

**Figure 2.**
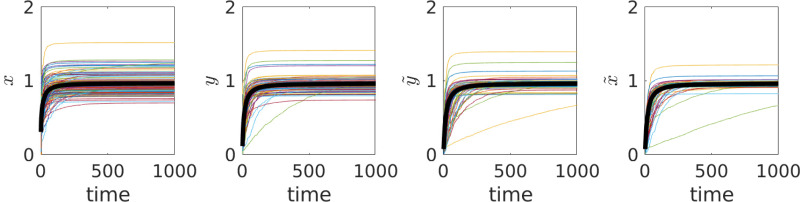
The dynamics of 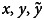 and 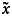 of individual players in the Coordination Game with no external influence observed in a single run of agent-based simulations. The thick black lines show the group averages. Group size *n* = 100. Parameters are chosen randomly and independently from certain distributions (as described in the Supporting Information) so that the mean value of *θ* is equal to 1. Initial values of 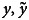 and 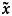 are chosen randomly and independently from a uniform distribution on [0, 0.1].

Equations (4) and (5) describe the joint dynamics of actions (*x*), attitudes (*y*) and beliefs (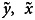). Numerical iterations of these equations show convergence to a stochastic equilibrium (see Figure 2 for an example to be considered in detail below). In the Supporting Information, I find an approximation for this equilibrium. Here I summarise what happens in several important special cases. For the rest of this paper, variables 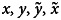 and *X* will specify the corresponding equilibrium values (rather than the dynamically changing values as above).

### No external influence; no variation in material payoffs

Assume that the external influence is absent (i.e. *A*_4_ = *C*_*i*3_ = *γ*_*i*_ = 0 for all *i*) and that there is no variation in material payoffs between individuals (so that coefficient *θ* is the same for all individuals). Then the system evolves to an equilibrium at which6
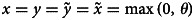
for all individuals. That is, with no variation in material costs and benefits, the population eventually becomes homogeneous in actions, attitudes and beliefs independently of the differences between individuals in all other parameters (i.e. *A*_*i*_, *α*_*i*_, *β*_*i*_). The value of *x* at equilibrium is the one maximising the material payoff.

### External influence only

If there are no material payoffs in the utility function (i.e. if all *D*_*i*_ = 0) while the external authority promotes action *G*, then at a long-term equilibrium7
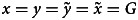
for each individual. That is, the population's actions, attitudes and beliefs are completely determined by the external influence and there is no variation between individuals.

### No external influence; variation in material payoffs

With variation in material benefits and costs between individuals (which is present in any realistic situation), one finds that the system evolves to an equilibrium state at which the average action8*a*
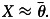
[Here and below the bar means the average over the whole population.] That is, at equilibrium the average action is the average of individual *θ*s which depend only on material payoffs. I also find that at equilibrium for each individual8*b*
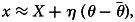
8*b*
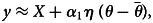
8*c*
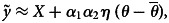
8*d*
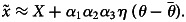


A composite parameter *η*, which depends on *B*s and *α*s, is defined by equation (S4c) in the Supporting Information. Parameters *θ*, *α*_1_, *α*_2_, *α*_3_ and *η* are individual specific while *X* and 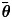 are the same for all individuals.

With no cognitive dissonance (i.e. if *α*_1_ = 0), 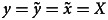, so that, the society becomes homogeneous in attitudes and beliefs while still exhibiting variation in actions *x*. Without the ‘theory of mind’ (i.e. if *α*_2_ = 0), 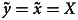, so that the society becomes homogeneous in beliefs while still exhibiting variation in actions *x* and attitudes *y*. Without logic constraints (i.e. if *α*_3_ = 0), 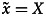, so that there will be no variation in second-order 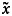 beliefs about actions. Note that if the correlation between 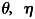 and the strength of cognitive factors *α*_1_, *α*_2_, *α*_3_ are low, the mean values of 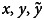 and 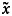 are all approximately equal to 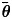. That is, on average individual preferences and beliefs align with actions.

One can also approximate the corresponding variances (see the Supporting Information). These approximations show that at equilibrium9
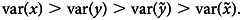
That is, the model predicts that the variation in actions will be the largest, followed by the variation in personal norms, followed by the variation in beliefs about norms of others, followed by the variation in beliefs about the action of others. A factor contributing to this pattern is that social influences act to align individual beliefs while differences in material payoffs are not affected by social influences and remain present. Similarly, the correlation with material benefits (characterised by parameter *θ*) will be the highest for individual actions *x*, followed by personal beliefs *y*, followed by normative expectations 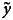 and empirical expectations 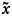 (see the Supporting Information). The predictions about the properties of long-term equilibria made in this section are testable.

### Examples

Next I illustrate my results using several games which have been extensively studied using methods of classical game theory, evolutionary game theory and behavioural economics. In experimental studies, the subjects are usually identical in terms of the expected costs and benefits of their actions. In contrast, in real life there is usually a lot of variation between individuals in these factors. Consequently, I will consider a group of *n* individuals who differ in various relevant characteristics such as their costs, benefits and/or valuation of the resource produced. (See S. Gavrilets 2015 for a review of models of collective action in heterogeneous groups.) I will also allow for differences between individuals in parameters characterising the effects of non-material factors.

In agent-based simulations, I will assign parameters *D*_*i*_, *A*_*i*_ of the utility function *u* and parameters *C*_*ij*_ specifying the dynamics of attitudes and beliefs randomly and independently from certain distributions. In my graphs, I will use an additional parameter *ɛ* which will vary from 0 to 1. I will scale parameters *A*_1_,…, *A*_4_ by multiplying them by *ɛ*. For example, with *ɛ* = 0 any normative effect in the utility function will be absent and individuals will behave according to standard evolutionary game theory assumptions. In contrast with *ɛ* = 1, the expected weight of each term in the utility function will be the same. Individuals will revise their actions and beliefs with probability 50% per individual per time step. I will also introduce small random errors during the update processes. I will compute the means and standard deviations of my variables at a long-term equilibrium, the Kendall rank correlation between them and *θ*, and the half-time *τ* of convergence to an equilibrium (defined as the time to reduce the distance to an equilibrium value by one half). My main focus will be on games with quadratic payoffs functions. However in the Supporting Information, I also consider several models with linear and quasi-linear payoff functions and a more complex example of a non-linear payoff function. Table S1 in the Supporting Information summarises the games I consider.

### Coordination game

Let individuals interact in randomly formed groups. Following Kuran and Sandholm (2008) (see also Andreoni et al., 2021), assume that each player pays a cost if his action deviates from the average action of the group. Without any additional factors, there is a line of equilibria in *x* that the groups can converge on. Further assume that each individual has a preferred action *θ*_*i*_ and pays a cost proportional to the square of the deviation from *θ*_*i*_. The corresponding (subjective) payoff function for individual *i* is10
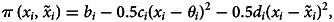
where parameter *b*_*i*_ is the maximum benefit, and *c*_*i*_ and *d*_*i*_ are parameters measuring the costs of deviation from the personally preferred action and from the mismatch with the partners’ actions, respectively. Here parameter *θ*_*i*_ defined by equation (3) is exactly *θ*_*i*_ of the payoff function (10).

*Evolutionary game theory* analysis. Let
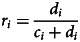
be the relative strength of conformity pressure for individual *i*. Assume that parameters *θ*_*i*_ and *r*_*i*_ are chosen randomly and independently from certain distributions. Then there is a single Nash equilibrium effort for individual *i* which can be approximated as 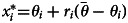, and the average effort of the group 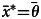 (see the Supporting Information).

#### General case

The average action predicted by my approach is the same: 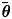. However the predictions for individual values 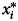 will differ between the two approaches (because *η* in equation 8b is different from *r*). Obviously, besides 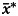 and 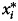, my model makes predictions for the expectations and variances of 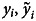 and 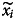.

**Figure 3.**
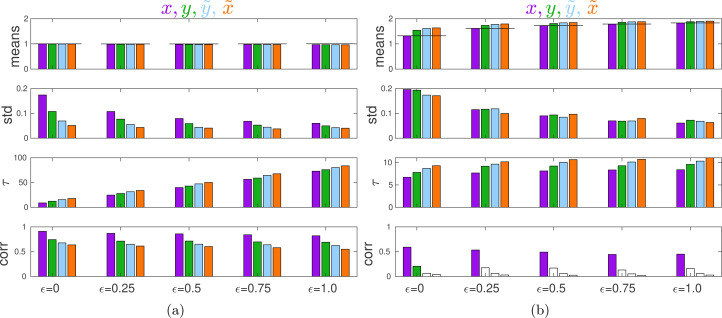
Properties of equilibria in the Coordination Game. (a) No external influence. (b) With external influence (*G* = 2). From top to bottom: mean, standard deviation, half-time of convergence to an equilibrium *τ*, and Kendall rank correlation with *θ* for *x* (purple), *y* (green), 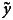 (blue) and 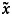 (orange), respectively. Bars with no colour mean that the corresponding correlations are statistically insignificant (at 0.05). The thin black lines show the theoretical predictions for *x*. Notice the difference between *y*-axis scales on graphs for *τ*. Parameter *ɛ* measures the weight of each normative factor relative to material payoffs in the utility function. Group size *n* = 100. Parameters *θ*_*i*_, *c*_*i*_, *d*_*i*_ are drawn from log–normal distributions with mean 1 and standard deviation 0.1, so that 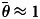. Statistics are calculated over the 100 last time steps over 40 independent runs each of length 1000 time steps.

Figure 3 illustrates the equilibria in this model found using agent-based simulations. The evolutionary game theory (EGT) predictions correspond to purple bars for *ɛ* = 0. The case of no external influence was modelled by setting all coefficients *A*_4_ and *C*_*i*3_ to zero. Figure 3a shows that, with no external influence:

- The mean values of 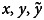 and 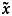 are close to 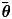 as predicted.
- Although with *ɛ* = 0 (leftmost set of bars), normative factors are absent from the utility function, variables 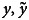 and 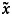 still evolve towards 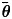 owing to the psychological processes modelled.
- The standard deviations and correlations with *θ* are in the order predicted – from the largest for *x* to the smallest for 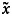.
- Increasing the strength *ɛ* of normative factors decreases within-group variation in all traits and delays convergence to an equilibrium.

Figure 3b shows that with external influence (with *G* = 2, so that the authority effectively asks individuals to double their efforts):

- Individuals respond to external influence by increasing their efforts, attitudes and beliefs towards *G* as *ɛ* increases with the mean of 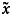 getting the closest to *G* and the mean of *x* lagging the most.
- Only *x* and, for *ɛ* = 0, *y* significantly correlate with *θ*.
- The time to convergence to the equilibrium is shorter than that without an external influence and does not depend much on *ɛ*.
- Even though with *ɛ* = 0 normative effects do not affect the utility function, mean actions are increased relative to the case of no external influence. This happens because the presence of an external influence increases individual beliefs 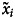 about the actions of others which in turn pushes them to increase their action *x*_*i*_ in order to coordinate better with groupmates.

### Public Goods game with quadratic personal costs

In this game, individuals make costly contributions to a total group effort *Z* the value of which is then multiplied by a constant factor *b*. The resulting amount *P* = *bZ* is then distributed back to the group members with *i*th individual getting value *v*_*i*_*P*, where *v*_*i*_ is a constant individual-specific parameter. For example, if each individual gets an equal share, *v*_*i*_ = 1/*n*. Following Calabuig et al. (2018), S. Gavrilets (2015), Esteban and Ray (2001) and McGinty and Milam (2013) assume that the cost to an individual is quadratic in their effort. In my framework, individual *i* making effort *x*_*i*_ predicts that his group effort will be 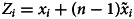. Then the estimated material payoff of individual *i* is11
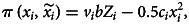
where *c*_*i*_ is an individual cost coefficient. Straightforward calculation then shows that *θ*_*i*_ = *v*_*i*_*b*/*c*_*i*_ which is just the benefit to cost ratio.

#### EGT analysis

The best response and the Nash equilibrium for the individual effort are equal to *θ*_*i*_ defined above.

#### General analysis

**Figure 4.**
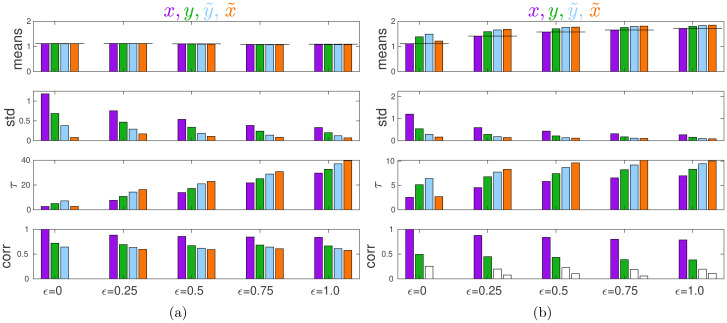
Properties of equilibria in the Public Goods game with quadratic costs. (a) No external influence. (b) With external influence promoting increased effort (*G* = 2). From top to bottom: equilibrium means, standard deviations, half-time of convergence to an equilibrium *τ* and Kendall correlation with *θ* for 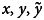 and 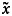, respectively. The thin black lines show the theoretical predictions for *x*. Parameter *ɛ* measures the importance of each of the normative factors relative to material payoffs. Group size *n* = 40. Parameters: *b*_*i*_ = 40for each *i*; parameters *c*_*i*_ are drawn from a log–normal distribution with mean 1 and standard deviation 0.1; parameters *v*_*i*_ are drawn from a broken stick distributions, so that 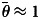. Statistics are calculated over 100 last time steps over 40 independent runs each of length 1,000 time steps.

Figure 4 illustrates the properties of equilibria in this model which are very similar to those in the Coordination game.

### Common Pool Resource game

In this game (Walker et al., 1990; Apesteguia and Maier-Rigaud, 2006), the production function shows a diminishing return in the group effort: *P* = *bZ* − 0.5*dZ*^2^, where *b* and *d* are constant parameters, and *Z* is the same as defined above. The individual payoff is12
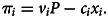
where *c*_i_ is the individual cost coefficient and the resource share going to individual *i* is proportional to their effort: *v*_i_=*x*_i_/*Z* as in the Tullock contest model (Konrad, 2009). In this model
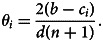


#### EGT analysis

In this model, Nash equilibria are 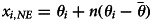. If all individuals have identical coefficients *c*_*i*_ = *c* and *b* > *c*, then the Nash equilibrium is 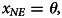 while the individual effort maximising the total group payoff is *x*_opt_ = (*b* − *c*)/*d*, that is, 2*n*/(*n* + 1) times smaller.

#### General analysis

**Figure 5.**
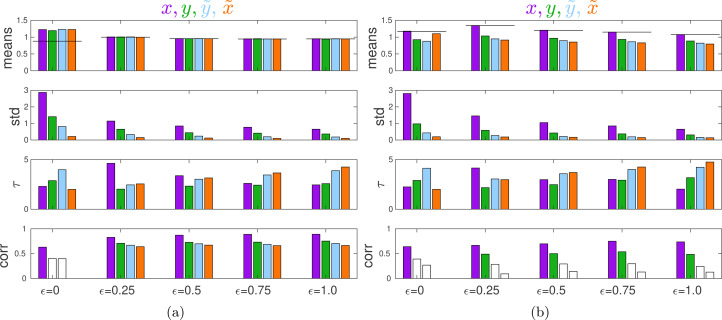
Properties of equilibria in the Common Pool Resources game. (a) No external influence. (b) With external influence promoting decreased, socially optimal effort *G* = 0.5. From top to bottom: equilibrium means, standard deviations, half-time of convergence to an equilibrium *τ*, and Kendall correlation with *θ* for 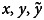 and 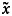, respectively. The thin black horizontal lines show the theoretical predictions for *x*. Parameter *ɛ* measures the importance of each of the normative factors relative to material payoffs. Group size *n* = 20. Parameters: *b*_*i*_ = 10 for each *i* while *c*_*i*_ and *d*_*i*_ are drawn from log–normal distributions with mean 1 and standard deviation 0.1 so that 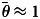. Initial values of 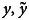 and 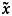 were chosen randomly and independently from a uniform distribution on [0, 0.1]. Statistics are calculated over 100 last time steps over 40 independent runs each of length 1000 time steps.

Figure 5a shows that with no external influence and positive *ɛ*, the general equilibrium patterns are similar to those in the two others games except that with *ɛ* = 0 the observed values exceed the predictions. This happens because of the non-equilibrium occasionally observed in this case (see the Supporting Information). The time to convergence is very short. With positive *ɛ*, all individual characteristics strongly correlate with the measure *θ* of material benefits.

With an external authority promoting a socially optimal individual effort *G* = *x*_opt_, group members actually increase rather than decrease their efforts (Figure 5b). In this game, the term *D*_1_ is proportional to the group size *n* which makes individual estimates of the expected payoff 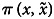 and, correspondingly, their best response *x* very sensitive to changes in 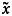 (see equations 2 and 4). If external authority promotes low efforts, individuals develop decreased expectations for 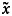 about the effort of others which in turn make them to believe that opportunistically increasing their own effort will be beneficial.

### Other games

In the Supporting Information, I consider a number of other games. A Tragedy of the Commons game with diminishing return, a game of the trade-offs between public and private production (Willinger and Ziegelmeyer, 1999, 2001; McGinty and Milam, 2013), and an ‘Us vs. Nature’ game (S. Gavrilets, 2015; S. Gavrilets and Richerson, 2017) show behaviour similar to that of the Public Goods game with quadratic costs (illustrated in Figure 4). In particular, in these four games individuals change their action in the direction promoted by an external authority. A Public Goods game with diminishing return (Anderson et al., 1998; Apesteguia and Maier-Rigaud, 2006) and a Tragedy of the Commons game with quadratic costs are similar to the Common Pool Resource game (illustrated in Figure 5). In particular, in these three games individuals can change their actions in the direction opposite to that promoted by an external authority. (In these games, the term *D*_1_ is linearly proportional to the group size *n*.)

I also consider several games with linear payoff functions (in which *D*_1_ = *D*_2_ = 0): the classical Dictator game and the Linear Public Goods as well as the Give-or-Take game (Bicchieri et al., 2020) and the Rule Following game (Kimbrough and Vostroknutov, 2016). In the EGT versions of these games, the Nash equilibrium effort is zero but the presence of an external influence can lead to positive efforts. Similar behaviour is exhibited by a continuous Prisoner's Dilemma game (Verhoeff, 1998) in which the payoff function is quasi-linear (i.e. *D*_2_ = 0 but both *D*_0_ and *D*_2_ are different from zero).

## Discussion

Here I have developed a unifying theoretical approach for modelling the dynamics of social interactions in situations where individuals’ personal norms and beliefs about others affect their own actions which in turn causes subsequent adjustments in norms and beliefs. My approach combines evolutionary game theory models focusing on material costs and benefits (Fudenberg and Tirole, 1992; Sandholm, 2010) and an adaptation of social influence models focusing on the dynamics of publicly expressed opinions (Watts, 2002; Friedkin et al., 2016; Redner, 2019) with novel modelling components capturing the dynamics of beliefs about others. In my approach, the publicly observable variables are individual actions while individual attitudes and beliefs are private and can only be guessed by others. Besides predicting individual and group behaviour, my models shed light on two other types of questions: which norms get internalised and which factors control beliefs about others.

### Individual characteristics

One of the goals of my approach was to understand the relative importance of different material, social and cognitive factors for individual behaviour and beliefs from the theoretical point of view. A major conclusion of my analysis is that some of these factors are much more important than others. My models predict that individual actions in social interactions, their attitudes (i.e. personal norms) and beliefs about others coevolve in a particular way. Specifically, the two most important factors in long-term dynamics are material payoffs and the influence of external authorities. In the absence of the latter, individual behaviour tends to evolve towards actions maximising their material payoffs while personal norms (attitudes) and beliefs about others exhibit coherence with individual actions. On longer time scales, variation in normative beliefs between individuals largely reflects variation in their material benefits and costs. My models thus predict that people have a tendency to internalise the ideas and beliefs that are most beneficial for their material well-being. In a sense, these modelling conclusions align with Marx’ postulate that ‘material life determines the social, political and intellectual life process in general’ (Marx, 1959).

At the same time, as stressed already by Aristotle, human nature is deeply social and political. Culture, social learning and conformity have played crucial roles since the origin of our species (Darwin, 1871; Richerson and Boyd, 2005; Henrich, 2015; Richerson et al., 2021). Therefore our actions, attitudes and beliefs are strongly affected by those of our peers as well as by external authorities (cultural, religious, political, administrative, etc). While peer influence largely works towards reducing variation between individuals, an external influence (or propaganda) can directionally shift actions, attitude and beliefs. This is a fact well known to politicians, religious leaders, cultural models, educators, marketing professionals and social media influencers. The resulting effects can be very positive or extremely negative from both individual and societal perspectives. My models predict how individual actions are dispersed around or shifted away from those maximising their personal material payoffs.

Under some conditions the effort of an authority to promote certain behaviour can backfire and cause an opposite effect. For example, the authority's messaging about the importance of participation in a collective action can develop higher expectations about the level of contributions of peers which then will lead individuals to opportunistically decrease their own costly effort. Alternatively, the authority's messaging about the need to reduce the consumption of a common resource can cause individuals to opportunistically increase their consumption. This is similar to situations captured by the Volunteer's Dilemma (Diekmann, 1985) – when individuals fail to perform an action they would benefit from because they expect others to volunteer.

In some of the models I considered, an external authority can cause individuals not only to perform actions detrimental for their material well-being but also to internalise preferences for such acts. My models can potentially be used to better understand obedience to authority such as that studied in Milgram's and Zimbardo's experiments (Milgram, 1963; Haney et al., 1973) or the effects of expected supernatural punishment for violating moral norms in moralising religions (Willard et al., 2020). My results may also be useful for better understanding of the causal effects of ‘institutional signals’ in developing better policies for social change, e.g. those stimulating pro-environment behaviour (Tankard and Paluck, 2016).

### Differences from EGT predictions

Standard EGT models aim to predict human behaviour solely from the expected material payoff. However, the growing understanding in behavioural economics is that certain normative factors must be considered to explain observed behaviour (Szekely et al., 2021; d’Adda et al., 2020; Andreoni et al., 2021; Loewenstein and Molnar, 2018; Fehr and Schurtenberger, 2018; Górges and Nosenzo, 2020; which is a fact well appreciated in social psychology). Allowing for certain normative factors in the utility function shifts the corresponding model predictions away from the Nash equilibrium based on the material payoffs. Recent work has offered empirically based ways to modify the utility function to explain apparently non-rational behaviour (i.e. deviations from Nash equilibria) of individuals in behavioural experiments (d’Adda et al., 2020; Andreozzi et al., 2020; Basić and Verrina, 2020; Kölle and Quercia, 2021).

My modelling framework allows one to treat and contrast the effects of multiple normative factors and psychological mechanisms on behaviour at once, thus generalising earlier work. Moreover one should also expect that the relative strengths of these factors and mechanisms will change as social interactions unfold. My approach offers a general theoretical way for describing these dynamics. It also allows for differences between individuals in various characteristics including psychology which are often disregarded in the EGT models.

Interestingly and importantly, allowing for changing attitudes and beliefs makes Nash equilibria of the EGT relevant again. Specifically my results show that in the absence of external authority, the average behaviour at a long-term equilibrium is exactly as predicted by the EGT (see also Calabuig et al., 2018). This gives some additional confidence in the robustness of some results/conclusions of the EGT. Besides choosing actions as predicted by the EGT on average, individuals are also predicted to develop attitudes and beliefs justifying (or matching) their behaviours.

However on short time scales and in the presence of an external authority, the two approaches will give very different predictions. Moreover even on long time scales, individual efforts can be smaller or larger than the EGT predictions and the distribution of individual efforts can be qualitatively different. For example, while some EGT models of collective action predict that only a single individual with the largest benefit-to-cost ratio will contribute to the group's effort (reviewed in S. Gavrilets, 2015), my models predict that there will be a large number of different contributors. The dynamics of attitudes and first- and second-order beliefs, which are at the core of my approach here, are outside of the scope of the EGT.

As noticed by an anonymous reviewer, the model's prediction that neither conformity with peers nor cognitive dissonance affects long-term average equilibrium behaviour may require us to reevaluate our assumptions about the adaptive function of these mechanisms, at least under conditions modelled here.

### Groups

My models allow for scaling up individual behaviour to group characteristics. In particular, within-group variation is predicted to be the largest for individual actions, followed by individual attitudes, followed by beliefs about attitude and actions. I also predict that a newly formed group (or a group encountering a new social situation) will go through a process of continuous reduction in these variances towards an equilibrium. This process can be interpreted as tightening of personal norms and normative and empirical expectations and can be studied experimentally (Szekely et al., 2021). Convergence to an equilibrium can be fast, although, of course, the actual time scale depends on parameters.

My variables 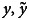 and 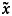 are closely related to the notion of personal, descriptive and injunctive (prescriptive) social norms (R. L. Cialdini et al., 1990; R. B. Cialdini and Goldstein, 2004; Bicchieri, 2006). In particular, variable *y* gives the personal norm of an individual. The average of 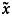 specifying the expected average behaviour of others defines the descriptive norm in the group. The average of 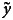 specifying the average belief of individuals about what others expect from them defines the injunctive norm (S. Gavrilets, 2020). My models shows how these norms become dynamically aligned as social interactions unfold. This process is a subject of recent experimental studies (Eriksson et al., 2015; Tworek and Cimpian, 2016; Lindstrom et al., 2018; Szekely et al., 2021).

My results show that pinning down theoretically the importance of each individual model component is hardly possible. For example, the average individual effort in the group at equilibrium depends on the weighted average of different types of individual parameters (e.g. see equation S8 in the Supporting Information). However this is expected given the complexity of social dynamics. Similar problems emerge and are successfully dealt with in other fields, e.g. statistical physics or genetics. I note that which forces and phenomena are most important in social behaviour is ultimately an empirical question.

### Tight and loose cultures

My theoretical results can be applied to cultural differences between different human groups. Empirical research shows that human cultures vary from very ‘tight’ to quite ‘loose’ in the degree to which they emphasise social norms and compliance with them (Pelto, 1968). The tight–loose (TL) differences can exist not only between different countries (Gelfand et al., 2011) but also within the same country, e.g. between 50 states in the US (Harrington and Gelfand, 2014) and between 31 provinces in China (Chua et al., 2019). The variation on the TL scale is also observed in non-industrial societies (Jackson et al., 2020). Gelfand et al. (2011), Harrington and Gelfand (2014), Jackson et al. (2020) and Roos et al. (2015) show with data that the TL variation can be explained in terms of the history of threats (e.g. environmental, internal and external warfare) faced by societies and the need to better coordinate collective actions under conditions of threat. Chua et al. (2019) confirm this interpretation but show that cultural tightness also correlates with tighter government control of areas of urbanisation and economic growth, with the strength of religious practices, and the extent of traditionality and group collectivism. Talhelm and English (2020) provided evidence that historically rice-farming societies have tighter social norms worldwide. They explained this by the fact that rice production was very labour intensive and required farmers to coordinate water use and develop strong norms for labour exchange. Using data on small-scale societies, Jackson et al. (2020) showed the importance of two additional factors: cultural complexity (sensu Murdock and Provost 1973) and kinship heterogeneity. Less complex societies and patrilocal societies (in which wives settle near their husband's parents) are tighter.

All of these analyses are correlational and therefore it cannot be claimed that the factors discussed there cause cultural tightness. However theoretical studies can provide support for causality. Roos et al. (2015) modelled cooperation in collective actions and showed that increasing the relative benefit of cooperation (which they interpreted as related to the level of the threat faced by the society) leads to a higher frequency of cooperative actions. The latter can be viewed as a measure of the strength of a (descriptive) cooperative norm.

Extending this work, my general approach allows one to study the effects of different factors not only on behaviour but also on individual attitudes and beliefs, both the average values and their distributions and correlations in the group. Next I discuss these effects within the context of the TL culture scale. In my model, the variation on this scale can be measured by the variances and coefficients of variation of 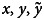 and 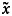.

### Social heterogeneity

My results show that in the absence of external influences, the most important factor in maintaining variation in actions, personal norms and beliefs is the variation in parameter *θ* measuring individual material costs and benefits (equation 3). Variation in *θ* is high if individuals differ in the roles they play in the society, their abilities, the compensation/valuation of the material benefit produced and the individual costs paid. This variation is directly related to social complexity of the society with simpler societies being expected to have less variation and, thus, stricter norms than more complex societies. My conclusion is thus in line with with the observations that urbanised areas have looser norms than rural areas (Harrington and Gelfand, 2014; Talhelm and English, 2020) and that more complex and heterogeneous societies have looser norms (Jackson et al., 2020).

### Societal threat

Behavioral response to a threat can often be just a rational change in the actions taken. For example, if cooperation becomes more profitable, its frequency is expected to increase as modelled in Roos et al. (2015). Societal threat will however also affect attitudes and beliefs, potentially making them more uniform and tightening culture (Szekely et al., 2021). There are several ways to introduce the effects of an environmental or social threat into my models. One is via a change in the payoff function *π*. In the Coordination Game, a threat can be modelled as an increase in the individual cost *d*_*i*_ of mismatch of the individual's action with the average action of peers. This would increase parameters *r*_*i*_ in that model, making the actions chosen more similar and consequently making all attitudes and beliefs more homogeneous. In other games with quadratic payoff function and in the Continuous Prisoner's Dilemma game, a societal threat can be modelled as a change in parameters *θ*_*i*_ measuring individual benefit-to-cost ratio. Although such a change will change the means and variances of actions, attitudes and beliefs, the corresponding coefficients of variation will not be affected. Societal threat can also increase the perceived cost of disapproval by peers *A*_2_, of non-conformity with peers’ action *A*_3_ and non-conformity with authority *A*_4_. Increasing these parameters will decrease *η*, reducing the variation in action, attitudes and beliefs, so that the society becomes more uniform.

### Propaganda effort

Societies also vary in the strength of the effort of political, religious, intellectual and other leaders and role models to promote certain types of behaviour. As discussed above, increasing the perceived cost *A*_4_ of non-conformity with authority will make the society more homogeneous. Similar effects can be achieved if the action *G* promoted by authority significantly deviates from 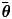, which can be viewed as a ‘natural’ optimum behaviour for the population. With sufficiently large values of *A*_4_, individual actions can shift towards *G*, ‘dragging’ individual attitudes and beliefs along and making them more uniform. For example, in China the strength of governmental control of provinces predicts norm tightness (Chua et al., 2019).

### Cultural variation

Data show significant cultural variation in conformity (Bond and Smith, 1996), cognitive dissonance (Heine and Lehman, 1997; Hoshino-Browne et al., 2005) and certain aspects of the Theory of Mind (Lillard, 1998; Lecce and Hughes, 2010; Heyes and Frith, 2014). Collectivistic cultures put special emphasis on conformity. In my model, such cultures would be characterised by increasing costs of non-conformity *A*_3_ and *A*_4_ and increasing parameters *β* and *γ* measuring the strength of social influence on attitudes and beliefs. Such increases will cause the society to become more uniform. Similar effects will be achieved by a decrease in the strength of cognitive dissonance (*α*_1_)and a reduced perception of logic constraints (*α*_3_), which would increase the ability to ‘doublethink’.

### Population size

In my models of collective action, I consider a single group the size of which enters explicitly only via parameter *D*_1_ and only in some models. Increasing the group size *n* increases *D*_1_ which will always decrease *θ* and the level of cooperation in the model because of increased free-riding. However the group size also enters implicitly because the perceived costs of disapproval by peers *A*_2_ and of non-conformity with peers’ action *A*_3_ are expected to increase with *n*. Therefore increasing population size is expected to make the culture tighter. This conclusion may appear to contradict the fact that urban areas show looser norms (see above). However, what this means is that the model predicts alternative pathways linking population size/density with norm tightness that have opposite effects. Which one is stronger is an empirical question.

### Differences in the subsistence style

Societies may differ in the types of social interactions that their members are most often involved in. For example, coordination and reciprocal exchange of labour was very important in rice production which has contributed to tighter cultures in rice-producing regions of the world relative to wheat-producing regions (Talhelm and English, 2020). As discussed above, the higher the cost of miscoordination, the tighter the society is predicted to be. Subsistence style also affects the extent to which people rely on social learning (Glowacki and Molleman, 2017).

Overall, my analysis provides theoretical support for a causal relationship between the factors just discussed and the extent of cultural tightness/looseness.

### Possible generalisations

My conclusions have important caveats though. First, they concern the expected average behaviour of the population. In any realistic situation one may expect the presence of individuals who will not be affected by certain factors included in my model. (Mathematically for such individuals, some of the corresponding coefficients *A*_*i*_, *D*_*i*_, *C*_*ij*_ will be equal to zero.) Second, my predictions mostly focus on long-term equilibria under the assumption of repeated interactions occurring according to a fixed set of rules. Predicting transient dynamics on short time scales is much more challenging. Third, my derivations assume that social interactions happen within a single constant group. An important future generalisation would be to consider interactions on a (dynamically changing) social network or in randomly formed groups. Also important is to consider the dynamics of beliefs represented by discrete rather than continuous variables (because it is know that their equilibria can be rather different; Zhong et al., 2012) and other types of utility function (1) allowing for multiple equilibria (Michaeli and Spiro, 2017). Additional potential generalisations include multidimensional extensions of the model (Converse, 1964; Friedkin et al., 2016; Kashima et al., 2021), more realistic models of learning (e.g. Bayesian learning, Khalvati et al. 2019) and strategy revision, equity concerns and learning from others’ performance. It would be interesting to use my models for studying political polarisation (Lees and Cikara, 2021) as well as the processes through which people change their social identity (Green, 2020).

### Model validation

My models can be validated using data from experiments or surveys. For example, the methods of experimental economics can be used to elicit beliefs about the actions and attitudes of others (d’Adda et al., 2020; Górges and Nosenzo, 2020; Gill and Rosokha, 2020; Andreozzi et al., 2020; Szekely et al., 2021). For example, d’Adda et al. (2020) measured subjects’ actions and beliefs corresponding to my variables 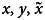 and 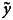 in a single round of the Dictator game while Szekely et al. (2021) did the same for a group of subjects playing a collective risk game (Milinski et al., 2008) over 28 rounds. Compliance with authority was studied in a Public Goods game (Silverman et al., 2014) and in the Joy of Destruction game (Karakostas and Zizzo, 2016). Importantly, because my main equations (e.g. equation 5) are linear, estimating the distributions of relevant parameters using (e.g. multilevel) regressions should be relatively straightforward. In experimental economics studies of social dilemmas it is common to classify subjects into different types such as altruists, free-riders and conditional cooperators (Fehr and Schurtenberger, 2018; Fehr and Fischbacher, 2004). Similar approaches can be used to study differences between individuals in their tendencies to change their personal norms and beliefs. In principle, it may be possible to compare quantitatively the relative strengths of cognitive factors (*α*s in my models), of learning from others (*β*s) and of complying with authority (*γ*s). Existing surveys that correlate different characteristics of societies with the tightness–looseness of their norms (Gelfand et al., 2011; Harrington and Gelfand, 2014; Chua et al., 2019; Jackson et al., 2020) as well as studies of how values and social preferences change over time (Tormos, 2020; Kiley and Vaisey, 2020; Böhm et al., 2021) offer additional opportunities to test my models.

People's attitudes and beliefs are important not only in social dilemmas as considered here but also in many other aspects of our life. They change dynamically throughout a person's life as a result of experiences (both personal and shared) and other psychological processes. They must be considered when scholars, practitioners or policymakers try to understand or predict social processes happening at different levels of our societies. The models developed here offer a way of doing it from the theoretical point of view. The challenge will be to integrate these models with empirical work.

## Research transparency and reproducibility

All data are in the manuscript. The Matlab code used is available upon request.

## Supplementary material

For supplementary material accompanying this paper visit https://doi.org/10.1017/ehs.2021.40. click here to view supplementary material
