# Supplementary material for: Coevolution of actions, personal norms and beliefs about others in social dilemmas
Source: Evol Hum Sci. 2021 Aug 19;3:e44. doi: 10.1017/ehs.2021.40 (PMC10427329; doi:10.1017/ehs.2021.40)
Supplement: Supplementary file 1 [file S2513843X21000402sup001.pdf]

Supplementary Information

Coevolution of actions, personal norms, and beliefs about others in social dilemmas

Sergey Gavrilets

Contents

Best response action **S1**

A single individual joining a large and stable social system **S2**

Equilibrium in a general case **S3**
- Quadratic payoff function with no external influence . . . S4
  - No variation in material costs and benefits . . . S4
  - Variation in material costs and benefits parameters . . . S4
- External influence only . . . S5
- Linear payoff function with an exogenous influence . . . S5

Games **S6**
- Numerical procedure . . . S6
- Coordination Game . . . S7
- Public Goods Game with quadratic personal costs . . . S9
- Public Goods Game with diminishing returns . . . S10
- Common Pool Resources Game . . . S13
- Tragedy of the Commons Game with quadratic costs . . . S15
- Tragedy of the Commons game with diminishing returns . . . S17
- Trade-offs between public and private production . . . S19
- Games with linear payoff functions: Dictator, Take-or-Give, Rule Obedience, and Public Goods . . . S21
- Continuous Prisoner's Dilemma game . . . S23
- "Us v. nature" game . . . S24

Best response action

The action x maximizing the utility function u can be found by computing the derivative of the utility function (1):

$$\begin{aligned}\frac{\partial u}{\partial x} =& D_0 - D_1\tilde{x} - D_2 x - A_1(x-y) - A_2(x-\tilde{y}) - A_3(x-\tilde{x}) - A_4(x-G),\\ =&(D_0 - D_1\tilde{x} + A_1 y + A_2\tilde{y} + A_3\tilde{x} + A_4 G) - (D_2 + A_1 + A_2 + A_3 + A_4)x.\end{aligned}$$

Solving the above equation for x gives us the best response action given a certain attitude y and beliefs $\tilde{x}$ and $\tilde{y}$. I will write it as

$$x_{\text{BR}} = \max(0, B_0 + B_1 y + B_2\tilde{y} + B_3\tilde{x} + B_4 G), \tag{S1a}$$

where

$$B_0 = \frac{D_0}{S}, B_1 = \frac{A_1}{S}, B_2 = \frac{A_2}{S}, B_3 = \frac{A_3 - D_1}{S}, B_4 = \frac{A_4}{S} \tag{S1b}$$

are re-scaled individual-specific parameters measuring the effects of material and nonmaterial forces on individual actions ($i = 0, 1, 2$ and 3), and

$$S = D_2 + \sum_{i=1}^{4} A_i \tag{S1c}$$

The above equation for x_{BR} naturally assumes that $S \neq 0$. In analogous evolutionary game theory models (in which all coefficients A_i are zero), S will be zero if $D_2 = 0$. In this case, the best response x_{BR} will be equal to a maximum (if $D_0 - D_1\tilde{x} > 0$) or 0 (if $D_0 - D_1\tilde{x} < 0$) possible value of x.

As an example, if one disregards all other forces involved in decision-making and focus only on material cost-benefit considerations (i.e. if all $A_i = 0$), the best response action will be

$$x_{\mathrm{BR}} = \frac{D_0 - D_1\tilde{x}}{D_2}. \tag{S2a}$$

If the individual believes that the average action of their social partners will always match their own action (i.e., $\tilde{x} = x$),

$$x_{\mathrm{BR}} = \frac{D_0}{D_1 + D_2}. \tag{S2b}$$

which is the definition of parameter θ (equation 3 of the main text).

Note that in standard evolutionary game theory (EGT) models using myopic best response, variable $\tilde{x}$ is replaced by the average action $\sum_{j\neq i} x_{j,prev}/(n-1)$ of their social partners which individuals know exactly.

A single individual joining a large and stable social system

Assume that an individual joins a society where the actions, attitudes and beliefs have already evolved to a certain stable distribution. Let the society be large enough so that the impact of a single additional individual on it is negligible. This will allow us to treat the average action of social partners X as constant. I am interested in how the individual's characteristics will change after joining the society. [Note that this model can be used for describing the subject's behavior when embedded in a group with bots acting according to a certain pre-programmed pattern.]

The attitude and beliefs of the focal individual will change according to recurrence equations (S3). Assume that they converge to an equilibrium $(x^*, y^*, \tilde{y}^*, \tilde{x}^*)$ at which $x^* > 0$. From equations (5) and using the fact that $\beta_i = 1 - \alpha_i - \gamma_i$ for all i, at this equilibrium:

$$y^* = X + \alpha_1(x^* - X) + \gamma_1(G - X) \tag{S3a}$$

$$\tilde{y}^* = X + \alpha_2(y^* - X) + \gamma_2(G - X) = X + \alpha_1\alpha_2(x^* - X) + (\alpha_2\gamma_1 + \gamma_2)(G - X), \tag{S3b}$$

$$\tilde{x}^* = X + \alpha_3(\tilde{y}^* - X) + \gamma_3(G - X) = X + \alpha_1\alpha_2\alpha_3(x^* - X) + (\alpha_3(\alpha_2\gamma_1 + \gamma_2) + \gamma_3)(G - X), \tag{S3c}$$

Substituting these into the best response equation (S1a) and solving for x,

$$
\begin{aligned}
x =& \frac{B_0 + B_4 G + (B_1 + B_2 + B_3 - B_1\alpha_1 - B_2\alpha_1\alpha_2 - B_3\alpha_1\alpha_2\alpha_3)X \dots}{1 - (B_1\alpha_1 + B_2\alpha_1\alpha_2 + B_3\alpha_1\alpha_2\alpha_3)} \\
& \frac{\dots + (G - X)\left[B_1\gamma_1 + B_2(\gamma_1\alpha_2 + \gamma_2) + B_3(\gamma_1\alpha_2\alpha_3 + \gamma_2\alpha_3 + \gamma_3)\right]}{\dots} \\
=& \frac{B_0 + B_4 G}{1 - (B_1\alpha_1 + B_2\alpha_1\alpha_2 + B_3\alpha_1\alpha_2\alpha_3)} \\
&+ \left(\frac{B_1 + B_2 + B_3 - 1}{1 - (B_1\alpha_1 + B_2\alpha_1\alpha_2 + B_3\alpha_1\alpha_2\alpha_3)} + 1\right) X \dots \\
&+ \frac{B_1\gamma_1 + B_2(\gamma_1\alpha_2 + \gamma_2) + B_3(\gamma_1\alpha_2\alpha_3 + \gamma_2\alpha_3 + \gamma_3)}{1 - (B_1\alpha_1 + B_2\alpha_1\alpha_2 + B_3\alpha_1\alpha_2\alpha_3)}(G - X).
\end{aligned}
$$

Therefore the equilibrium value of x can be written as

$$x^* = \delta + (1 - \eta)X + \xi(G - X), \tag{S4a}$$

where

$$
\begin{aligned}
\delta =& \frac{B_0 + B_4 G}{1 - (B_1\alpha_1 + B_2\alpha_1\alpha_2 + B_3\alpha_1\alpha_2\alpha_3)}, && \text{(S4b)} \\
\eta =& \frac{1 - B_1 - B_2 - B_3}{1 - (B_1\alpha_1 + B_2\alpha_1\alpha_2 + B_3\alpha_1\alpha_2\alpha_3)}, && \text{(S4c)} \\
\xi =& \frac{B_1\gamma_1 + B_2(\gamma_1\alpha_2 + \gamma_2) + B_3(\gamma_1\alpha_2\alpha_3 + \gamma_2\alpha_3 + \gamma_3)}{1 - (B_1\alpha_1 + B_2\alpha_1\alpha_2 + B_3\alpha_1\alpha_2\alpha_3)} && \text{(S4d)}
\end{aligned}
$$

Note that $\xi = 0$, if propaganda by the external authority does not affect individual attitude and beliefs (i.e. all $\gamma_i = 0$). Correspondingly, the deviation of x^* for a focal individual from X can be written as

$$x^* - X = \delta - \eta X + \xi(G - X). \tag{S5}$$

From here it is straightforward to find equilibrium values of $y, \tilde{y}$ and $\tilde{x}$ using equations (S3). Note that only non-negative values of $x^*, y^*, \tilde{x}^*, \tilde{y}^*$ and X make sense within my framework.

Equilibrium in a general case

In the general case, all n individuals will be updating their attitudes and beliefs and the average efforts of peers X will be changing in time. However one still can use equation (S4a) to approximate the equilibrium. Specifically, summing up across all individuals and equating the average values of x and X, one finds that at equilibrium

$$X^* = \frac{\overline{\delta} + G\overline{\xi}}{\overline{\eta} + \overline{\xi}}, \tag{S6a}$$

where the bar means the average over the group.

In some of the models I consider in the main text, only a subset of individuals, typically with the largest benefit-to-cost ratios and/or most affected by external influences, make

contribution at equilibrium, while others free-ride. In such situations, to predict the average group effort one needs to sum up equations (S4a) only over a subset L of individuals making positive efforts. Equation (S6a) then takes a form

$$X^* = \frac{\sum_L \delta + G \sum_L \xi}{n - l + \sum_L \eta + \sum_L \xi}, \tag{S6b}$$

where l is the number of contributing individuals. In principle, one can find the individuals who make positive contributions at equilibrium using an iterative procedure similar to that in ref.Gavrilets and Fortunato (2014). I leave this for future work.

Knowing X^* allows us to find the equilibrium values of $x, y, \tilde{y}, \tilde{x}$ for each individual. Next I consider some special cases.

Quadratic payoff function with no external influence

Assume that external influence is absent so that $B_4 = \gamma_1 = \gamma_2 = \gamma_3 = 0$. Then $\xi = 0$. Note that in this case, the numerator in the equation for δ is D_0/S while that in the equation for η is $1 - (B_1 + B_2 + B_3) = (D_1 + D_2)/S$. Both equations have the same denominator. This implies that $\delta(D_1 + D_2) = \eta D_0$.

No variation in material costs and benefits

Assume that there is no variation in coefficients D_0, D_1 and D_2 between individuals. Then I find that

$$X^* = \frac{\overline{\delta}}{\overline{\eta}} = \frac{D_0}{D_1 + D_2} = \theta. \tag{S7}$$

That is, the average action is the action predicted if nonmaterial forces are neglected (see equation S2a). Therefore, $\delta - \eta X^* = 0$, so that $x^* = X^*$. From equations (S3), one concludes that $y^* = \tilde{y} = \tilde{x} = \theta$ for all individuals, which is equation (6) of the main text. That is, with no variation in material costs and benefits, the group will converge to a state with identical actions, attitudes, and beliefs.

Variation in material costs and benefits parameters

Allowing for variation in D_0, D_1 and D_2 and approximating the ratio of expectations $\frac{\overline{\delta}}{\overline{\eta}}$ by the expectation of ratio $\overline{\beta/\eta}$ I find that

$$X^* = \frac{\overline{\delta}}{\overline{\eta}} \approx \overline{\delta/\eta} = \overline{\frac{D_0}{D_1 + D_2}} = \overline{\theta}, \tag{S8a}$$

which is equation (8a) of the main text. That is, the average action is approximately the average of actions predicted if nonmaterial forces are neglected (see equation S2a).

Using equations (S5) and (S3), I find that at equilibrium for each individual

$$x^* \approx X^* + \eta\ (\theta - \overline{\theta}), \tag{S8b}$$

$$y^* \approx X^* + \alpha_1 \eta\ (\theta - \overline{\theta}), \tag{S8c}$$

$$\tilde{y}^* \approx X^* + \alpha_1 \alpha_2 \eta\ (\theta - \overline{\theta}), \tag{S8d}$$

$$\tilde{x}^* \approx X^* + \alpha_1 \alpha_2 \alpha_3 \eta\ (\theta - \overline{\theta}). \tag{S8e}$$

which are equations (8b,c,d,e) of the main text. With no cognitive dissonance (i.e. if $\alpha_1 = 0$), $y^* = \tilde{y}^* = \tilde{x}^* = X^*$. Without the "theory of mind" (i.e. if $\alpha_2 = 0$), $\tilde{y}^* = \tilde{x}^* = X^*$. Without beliefs dissonance (i.e. if $\alpha_3 = 0$), $\tilde{x}^* = X^*$. Note that mean values of $x^*, y^*, \tilde{y}^*$ and $\tilde{x}^*$ are all approximately equal to X^* if the correlation between θ, η and the strength of cognitive factors $\alpha_1, \alpha_2, \alpha_3$ is low. Assuming independence of θ and η, the corresponding variances are approximately

$$\mathrm{var}(x) = \mathrm{var}(\theta)\ \overline{\eta^2}, \tag{S9a}$$

$$\mathrm{var}(y) = \mathrm{var}(\theta)\ \overline{(\alpha_1 \eta)^2}, \tag{S9b}$$

$$\mathrm{var}(\tilde{y}) = \mathrm{var}(\theta)\ \overline{(\alpha_1 \alpha_2 \eta)^2}, \tag{S9c}$$

$$\mathrm{var}(\tilde{x}) = \mathrm{var}(\theta)\ \overline{(\alpha_1 \alpha_2 \alpha_3 \eta)^2}, \tag{S9d}$$

where $\mathrm{var}(\theta)$ is the variance of θ's in the group. Because $\alpha_1, \alpha_2, \alpha_3 < 1$, all this implies that

$$\mathrm{var}(x) > \mathrm{var}(y) > \mathrm{var}(\tilde{y}) > \mathrm{var}(\tilde{x}), \tag{S10}$$

which is inequality (9) of the main text. That is, my model predicts that the variation in actions (and deviation from the mean) will be the largest, followed by the variation in personal norms, followed by the variation in beliefs about norms of others, followed by the variation in beliefs about the action of others. Similarly, the correlation with material benefits (characterized by parameter θ) will be the highest for personal beliefs y, followed by normative expectations $\tilde{y}$, and empirical expectations $\tilde{x}$. These are testable predictions.

External influence only

If there are no material payoffs in the utility function, i.e. if $D_0 = D_1 = D_2 = 0$, straightforward calculation shows that $\sum B_i = 1$ and that $\eta - \delta/G = 0$ for each individual. Therefore, using equations (S3)-(S4a) for each individual,

$$x^* = y^* = \tilde{y} = \tilde{x} = G, \tag{S11}$$

which is equation (7) of the main text. That is, the population's actions, attitudes, and beliefs at long-term equilibrium are completely determined by the the external influence. There will be no variation between individuals.

Linear payoff function with an exogenous influence

Here I assume that $D_1 = D_2 = 0$ for all individuals while there is variation between individuals in D_0. The corresponding game-theoretic models neglecting nonmaterial factors

predict a simple behavior: individuals will make the maximum possible effort (if $D_0 > 0$) or no effort (if $D_0 < 0$). I will assume that $D_0 < 0$ which is the case in several games I consider below. With nonmaterial factors added but still with no external influence (i.e. $A_4 = 0$), individuals' actions, attitudes and beliefs will converge to 0.

Assume there is an external authority promotes a positive effort G. In this case, using equations (S1a) and (S1c) I find that $S = \sum_{i=1}^{4} A_i, \sum_{i=1}^{4} B_i = 1$, and that $\delta = (\kappa + G)\eta$, where

$$\kappa = D_0/A_4 \tag{S12}$$

is a measure of the strength of material forces relative to that of external influence. Then the average effort at equilibrium can be approximated as

$$X = G - \frac{\overline{\kappa\eta}}{\bar{\eta} + \bar{\xi}}. \tag{S13}$$

where $\kappa = |D_0|/A_4$ is a measure of the strength of material forces relative to that of external influences and the composite parameters ξ (defined in the SI) is non-negative. If the effect A_4 of an external authority is large enough, κ is small and the average group effort will be close to G.

One can now find the equilibrium values of $y, \tilde{x}$ and $\tilde{y}$ from equations (S3).

Games

Numerical procedure

In numerical simulations used to illustrate my results, I used the following procedure for generating parameter values. I start by assigning parameters D_0, D_1, D_2 by drawing numbers randomly and independently from certain distributions (specified below). Then I assign parameters $A_1, \ldots, A_4$ of the utility function (1) using a two-step procedure. At the first step, I choose them randomly and independently from a "broken stick distribution" on a unit interval (MacArthur, 1957). Then I multiply these numbers by a parameter ε which will vary from 0 to 1. With $\varepsilon = 0$, any normative effect in the utility function will be absent and individuals will behave according to standard evolutionary game theory assumptions. In contrast with $\varepsilon = 1$, the expected values of each of parameters A_i will be the same as that of D_2 (in models with $D_2 \neq 0$) or D_0 (in models with $D_2 = 0$). That is, with $\varepsilon = 1$, the expected weight of each term in the utility function (1) will be the same. Finally I draw parameters C_{ij} randomly and independently from a broken stick distribution on interval $[0, 0.1]]$. Initial values of $x, y, \tilde{y}$ and $\tilde{x}$ are drawn randomly and independently form a uniform distribution on $[0, 0.1]$. At each round, each individual revises its effort with probability 0.5. [In some simulations, after each update I have perturbed the dynamic variables by small random errors drawn from a uniform distribution on $[-\sigma, \sigma]$. The effects of such random noise are intuitive. Therefore for clarity, I removed it from the simulations illustrated in all figures.]

The graphs in the main text and below show the average values of $x, y, \tilde{y}, \tilde{x}$ observed in numerical simulations. The thin black lines show the theoretical predictions for x computed using approximation (S6a). However in two games - the Public Goods Game with quadratic personal costs and the Tragedy of the Commons Game with quadratic costs - if $\varepsilon = 0$ (i.e., only material payoffs present), the theoretical prediction is given by $\max(\theta_i)$ (see below).

Table S1: Production function P (or p_i) and expected payoffs $\pi(x_i, \tilde{x}_i)$ in different games. Games with quadratic payoff functions: Coordination, Public Goods Game (PGG) with quadratic costs, PGG with diminishing return, Common Pool Resource (CPR), Tragedy of the Commons (TC) with quadratic costs, and TC with diminishing return. Games with linear payoff functions: Dictator, Give-or-Take, Rule Following, and Linear PGG. Game with quasi-linear payoff function: continuous Prisoner's Dilemma. Nonlinear game: "us vs. nature" game. In all collective action games, the expected group effort is $Z = x + (n-1)\tilde{x}_i$. An empty entry in the table means that in the corresponding game the corresponding function or parameter is not defined. Parameters with subscript i (e.g., b_i, c_i, d_i, r_i, v_i) are specific for individuals. Parameters without subscripts (e.g.., b, d, R) are the same for all individuals. R and r_i are the endowments. Note that variable P in collective action games is the production function while in the Prisoner's Dilemma game, P_i is the punishment payoff.

Game	Production P/p_i	Expected payoff π_i
Coordination		$b_i - 0.5c_i(x_i - \theta_i)^2 - 0.5d_i(x_i - \tilde{x}_i)^2$
PGG w/ quadratic costs	$P = bZ$	$v_iP - 0.5c_ix_i^2$
PGG w/ diminishing return	$P = bZ - 0.5dZ^2$	$v_iP - c_ix_i$
CPR	$P = bZ - 0.5dZ^2$	$\frac{x_i}{Z}\ P - c_ix_i$
TC w/ quadratic costs	$p_i = b_ix_i$	$p_i - 0.5c_iZ^2$
TC w/ diminishing return	$p_i = b_ix_i - 0.5d_ix_i^2$	$p_i - c_iZ$
Public vs. private production	$P = bZ - 0.5dZ^2$	
	$p_i = b_iz_i - 0.5d_iz_i^2$	$v_iP + p_i$, where $z_i + x_i = r_i$
Dictator		$R - x_i$
Give-or-Take		$R - x_i$
Rule Following		$R - x_i$
Linear PGG	$P = bZ$	$v_iP - c_ix_i$
Continuous PD		$x_i\tilde{x}_iR_i + x_i(1-\tilde{x}_i)S_i \ldots$
		$+(1-x_i)\tilde{x}_iT_i + (1-x_i)(1-\tilde{x}_i)P_i$
"Us vs. nature"	$P = b\frac{Z}{Z+Z_0}$	$v_iP - c_ix_i$

Table S1 summarizes the games I will consider. For each game, I will: a) define the payoff function $\pi(x, \tilde{x})$ and identify the corresponding θ value, b) identify the Nash equilibrium in the corresponding evolutionary game theory (EGT) model, and c) show results of agent-based simulations illustrating individual and group characteristics and compare them with my approximations and EGT predictions. In the EGT versions of these games, individuals will use best response to maximize their payoff. The corresponding payoff functions will be the same as specified in Table 1 except that the term $\tilde{x}_i$ (empirical expectation of peers' action) will be replaced by the average action $\overline{x}_{i,prev}$ of groupmates at the previous time step as is usually done in best-response modeling.

Coordination Game

I assume that individuals interact randomly in groups. Each individual has a preferred action but each player also pays a cost if his action deviates from the average action of the group (Kuran and Sandholm, 2008, Andreoni *et al.*, 2021). The corresponding (subjective)

expected payoff function can be written as

$$\pi(x_i, \tilde{x}_i) = b_i - 0.5c_i(x_i - \theta_i)^2 - 0.5d_i(x_i - \tilde{x}_i)^2, \tag{S14}$$

where $\theta_i \geq 0$ is the preferred action of individual i, $\tilde{x}_i$ is the expected average action, b_i is the maximum benefit, and c_i and d_i are parameters measuring the costs of deviation from the personally preferred action and from the mismatch with the partners' actions. For this game, $d\pi/dx_i = -c_i(x_i - \theta_i) - d_i(x_i - \tilde{x}_i)$. Therefore $D_0 = c_i\theta_i, D_1 = -d_i, D_2 = c_i + d_i$. Parameter θ_i defined in equation (3) is exactly θ_i of the payoff function π.

EGT analysis. In the EGT version of this game, individuals will aim to maximizes the payoff function (S14) in which the term $\tilde{x}_i$ is substituted by the average action $\overline{x}_{i,prev}$ of groupmates at the previous time step. I will simplify my analysis by assuming that the groups size is large enough so that the effect of any single individual on the average is negligible. In this case, all $\overline{x}_{i,prev}$ values will approximately be the same, so that I can drop the subscript i.

Computing the derivative $\partial\pi/\partial x_i$, I find that the best response action for individual i is

$$x_{i,\mathrm{BR}} = (1 - r_i)\theta_i + r_i\overline{x}_{prev}, \tag{S15a}$$

where

$$r_i = \frac{d_i}{c_i + d_i}$$

is the relative strength of conformity pressure.

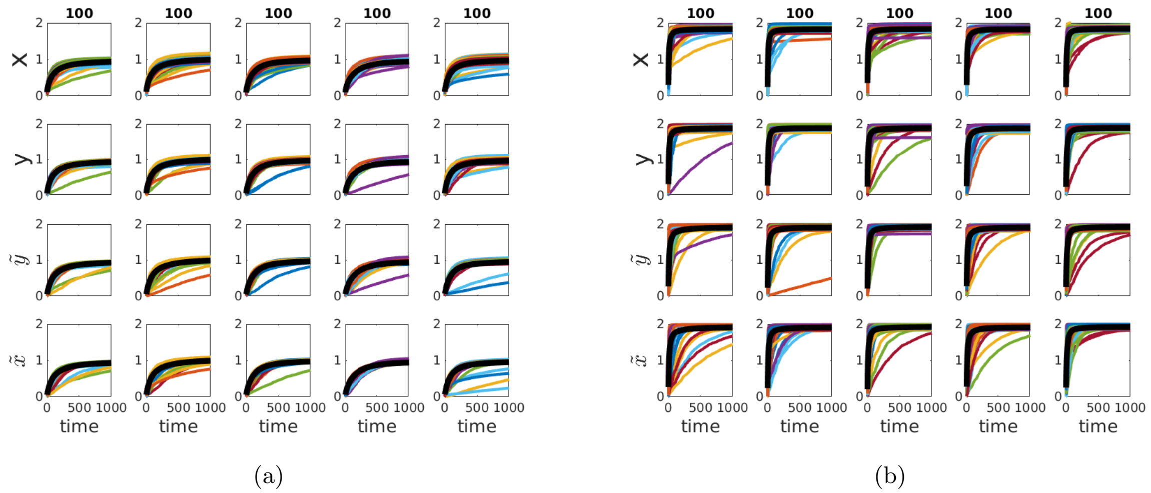

Figure S1: Examples of coevolutionary dynamics in the Coordination Game corresponding to Figure 3 of the main text. (a) Five runs with no external influence. (b) Five runs with external influence with $G = 2$. Different colors show different individuals. The thick black lines show the group averages. Group size $n = 100, \varepsilon = 1$. The numbers on top show the number of contributing individuals at the last time step. Other parameters: individual values of θ_i, c_i, d_i are drawn randomly and independently from lognormal distributions with mean 1 and standard deviation 0.1. Initial values of $y, \tilde{y}$ and $\tilde{x}$ were chosen randomly and independently from a uniform distribution on $[0, 0.1]$.

Assume that parameters θ_i and r_i are distributed in the group independently. Then the

average individual effort at (Nash) equilibrium is

$$\overline{x}^* = \overline{\theta}, \tag{S15b}$$

while the equilibrium effort for individual i can be written as

$$x_i^* = \theta_i + r_i(\overline{\theta} - \theta_i). \tag{S15c}$$

Here and below bars mean the average over the group.

General analysis. Figure 3 in the main text summarizes my results for this model. Figure S1 shows sample trajectories corresponding to $\varepsilon = 1$ (i.e. when all components of the utility function (1) are of similar order).

Public Goods Game with quadratic personal costs

In this game, individuals make costly contributions x_i to a common group effort Z the value of which is then multiplied by a constant factor b. The resulting amount $P = bZ$ is then distributed back to the group members with ith individual getting value v_iP. Following Esteban and Ray (2001), McGinty and Milam (2013), Gavrilets (2015), Calabuig *et al.* (2018), assume that the cost to an individual is quadratic in their effort. Then the expected material payoff of individual i making effort x_i given the expectation that the groupmates will make an average effort $\tilde{x}_i$ is

$$\pi(x, \tilde{x}) = v_i bZ - 0.5 c_i x_i^2, \tag{S16}$$

where c_i is the individual cost coefficient and the expected total group effort $Z = x_i + (n-1)\tilde{x}$.

One finds that $d\pi_i/dx_i = bv_i - c_i x_i$ so that $D_{0,i} = bv_i, D_{1,i} = 0, D_{2,i} = c_i$, and

$$\theta_i = \frac{bv_i}{c_i}. \tag{S17}$$

is just the benefit to cost ratio.

EGT analysis. In the EGT version of this model, the term $(n-1)\tilde{x}$ in the expression for Z will be substituted by the sum of efforts of groupmates at the previous time step, $Z^-_{i,prev} = \sum_{j \neq i} x_{j,prev}$. Then, the best response and the Nash equilibrium for individual effort is

$$x_{i,\mathrm{BR}} = x_{i,\mathrm{NE}} = \theta_i.$$

If all individuals have identical coefficients $v_i = 1/n$ and $c_i = c$, then the Nash equilibrium is

$$x_{\mathrm{NE}} = \frac{b}{nc}$$

while the effort maximizing the total group payoff is

$$x_{\mathrm{opt}} = \frac{b}{c},$$

that is, n times bigger.

General analysis. Figure 4 in the main text illustrates the general patters in this model.

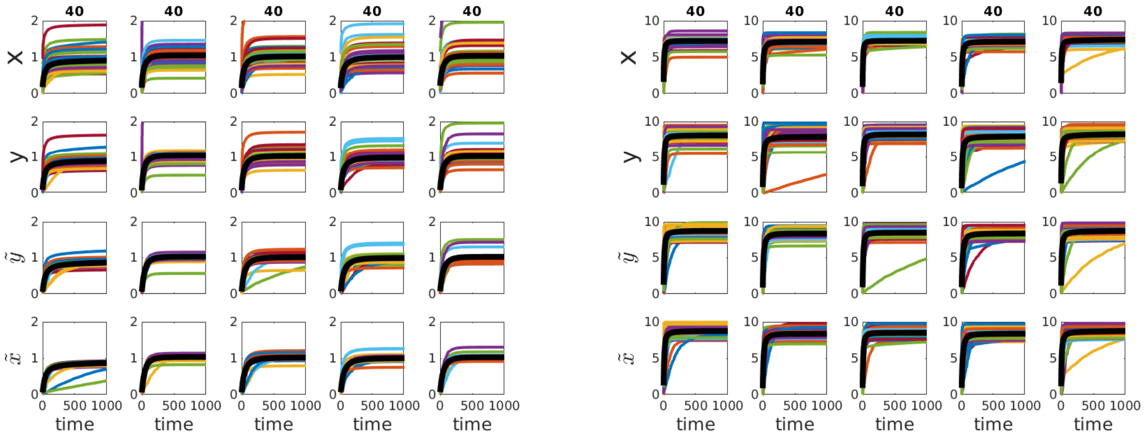

(a) (b)

Figure S2: Examples of coevolutionary dynamics in the Public Goods Game with quadratic costs. (a) Five runs with no external influence. (b) Five runs with external influence with $G = 10$. Different colors show different individuals. The thick black lines show the group averages. Group size $n = 20, \varepsilon = 1$. The numbers on top show the number of contributing individuals at the last time step. Other parameters: $b = n$, individual values of c_i are drawn randomly and independently from lognormal distributions with mean 1 and standard deviation 0.1, values of v_i are drawn from a broken stick distribution on interval $[0, 1]$. Initial values of $y, \tilde{y}$ and $\tilde{x}$ were chosen randomly and independently from a uniform distribution on $[0, 0.1]$.

Figure S2 shows sample trajectories of the general model corresponding to Figure 4 with $\varepsilon = 1$.

Public Goods Game with diminishing returns

In this game (Anderson *et al.*, 1998, Apesteguia and Maier-Rigaud, 2006), the production function shows a diminishing return in the group effort X:

$$P = bZ - 0.5dZ^2. \tag{S18a}$$

and the individual payoff is

$$\pi_i = v_i P - c_i x_i. \tag{S18b}$$

Here, $d\pi_i/dx_i = v_i(b - d(x_i + (n-1)\tilde{x}) - c_i$. Therefore $D_{0,i} = v_i b - c_i, D_1 = v_i d(n-1), D_2 = v_i d$, and

$$\theta_i = \frac{b - c_i/v_i}{dn}. \tag{S18c}$$

EGT analysis. In the EGT version of this game, the term $(n-1)\tilde{x}$ in the expression for $d\pi_i/dx_i$ will be substituted by the previous total effort $Z^-_{prev,i}$ of ith individual's peers. The best response effort is

$$x_{\mathrm{BR},i} = \max\left(0, n\theta_i - Z^-_{prev.i}\right).$$

In the symmetric version of this game when all coefficients are the same (i.e. $c_i = c$ and $v_i = 1/n$ so that all θ_i are the same), the Nash equilibrium for the total group effort is

$$Z_{\mathrm{NE},sym} = n\theta = \frac{b - cn}{d}.$$

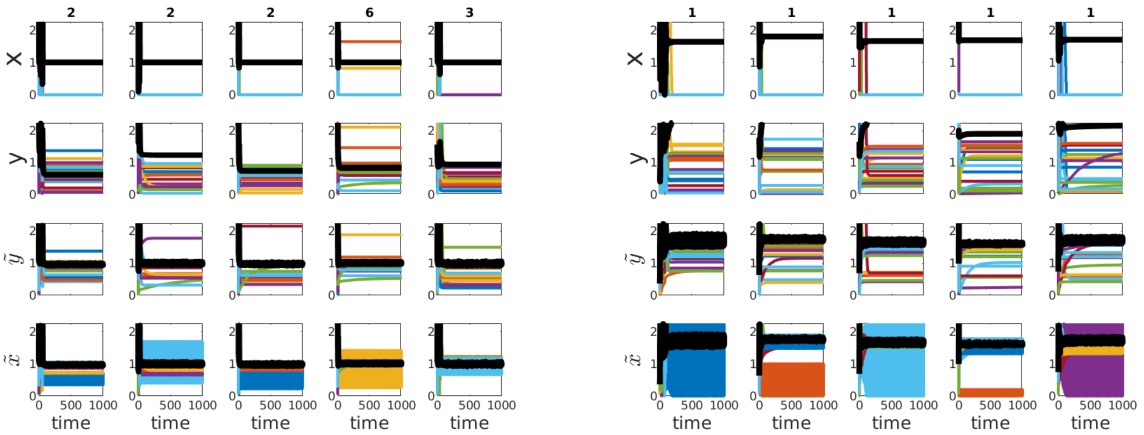

(a) (b)

Figure S3: Examples of best response dynamics in the Public Goods Game with diminishing returns and group size $n = 20$ and $\varepsilon = 0$. (a) Five run in the symmetric model with $v_i = 1/n$. (b) Five runs in the asymmetric model with v_i chosen randomly from a broken stick distribution. The numbers on top show the number of contributing individuals at the last time step. The appearance of nonlinear dynamics in a linear system may appear strange. However because of the truncation used to avoid negative values, the system effectively becomes nonlinear.

In contrast, the total group effort maximizing the total group payoff is

$$Z_{\text{opt}} = \frac{b-c}{d}.$$

Individual contributions can take any values as long as they sum up to $Z_{\text{NE},sym}$. Numerical agent-based simulations using myopic best response show that the system converges to this equilibrium, sometimes in a non-monotonic way; at this equilibrium the effort $Z_{\text{NE},sym}$ is supplied by one or few individuals (see Figure S3a).

In the asymmetric case, when values of v_i and c_i differ between individuals, the system evolves to an equilibrium at which only a single individual with the smallest value of c_i/v_i will make an effort (see Figure S3b). This effort, which is also the total group effort, is

$$Z_{\text{NE},asym} = \max(\theta_i).$$

General analysis. Figure S4 summarizes the properties of this model. The dynamics observed in agent-based simulations are often non-equilibrium (see Fig. S5). What happens is that a small number of individuals with sufficiently large values of θ_i are making large efforts while the rest of the population free-ride. For some individuals, contributions change in a cyclical or chaotic manner. The appearance of nonlinear dynamics in a linear system may appear strange. However because of the truncation used to avoid negative values of my variables, the system effectively becomes nonlinear.

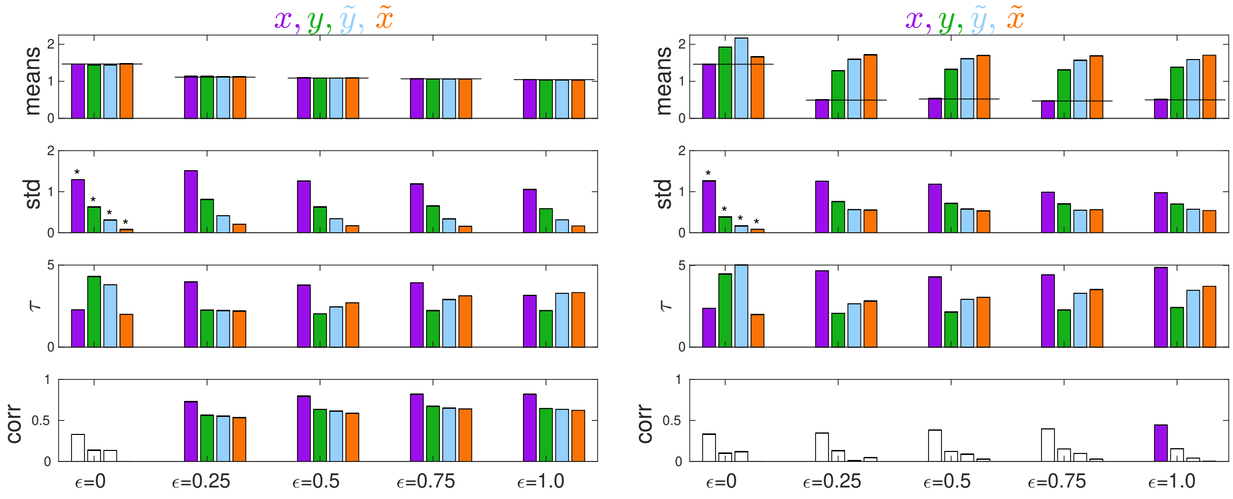

(a) (b)

Figure S4: Properties of equilibria in the Public Goods game with diminishing return. (a) No external influence. (b) With external influence with $G = 3$. From top to bottom: equilibrium means, standard deviations, half-time of convergence to an equilibrium τ, and Kendall correlation with θ for $x, y, \tilde{y}$ and $\tilde{x}$, respectively. Bars with no color mean the corresponding correlations are statistically insignificant (at 0.05). The thin black horizontal lines show the theoretical predictions for x. Parameter ε measures the importance of each of the normative factors relative to material payoffs. Group size $n = 20$. Other parameters $b_i = 2n, c_i = d_i = 1$ for all i while parameters v_i are drawn from a broken stick distribution. Initial values of $y, \tilde{y}$ and $\tilde{x}$ were chosen randomly and independently from a uniform distribution on $[0, 0.1]$. The stars on top of the bars for $\varepsilon = 0$ mean that the actual values of standard deviations are 5 times larger than shown. Statistics are calculated over 100 last time steps over 40 independent runs each of length 1,000 time steps.

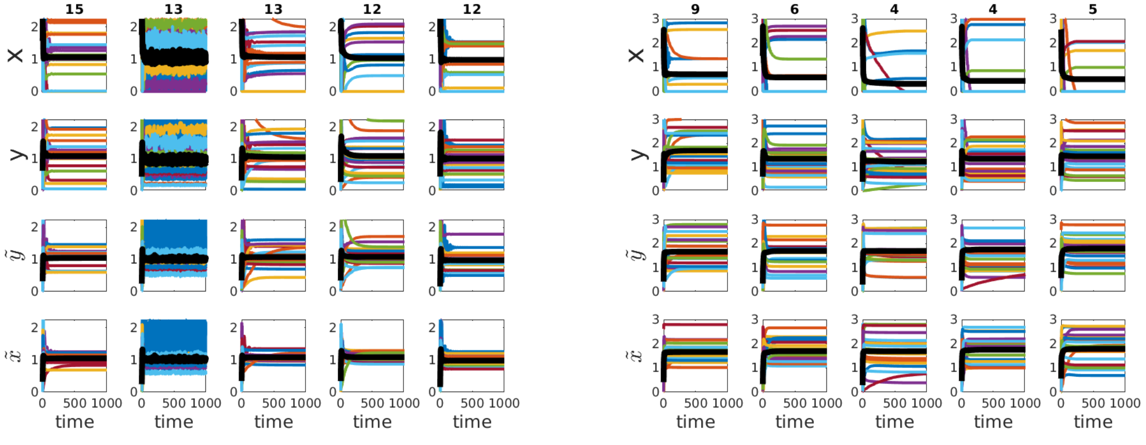

(a) (b)

Figure S5: Examples of coevolutionary dynamics in the Public Goods Game with diminishing returns corresponding to Figure S4. (a) Five runs with no external influence. (b) Five runs with external influence with $G = 3$. Group size $n = 20$, $\varepsilon = 1$. Different colors show different individuals. The thick black lines show the group averages. The numbers on top show the number of contributing individuals at the last time step. The appearance of nonlinear dynamics in a linear system may appear strange. However because of the truncation used to avoid negative values of dynamic variables, the system effectively becomes nonlinear.

Common Pool Resources Game

In this game (Walker *et al.*, 1990, Apesteguia and Maier-Rigaud, 2006), the production function shows a diminishing return in the group effort $Z = x_i + (n-1)\tilde{x}_i$:

$$P = bZ - 0.5dZ^2. \tag{S19a}$$

the individual cost is linear in effort x_i, and the individual payoff is

$$\pi_i = v_i P - c_i x_i \tag{S19b}$$

as in the Public Goods Game with diminishing returns considered above. However here valuation v_i is not a constant but rather depends on the individual's effort:

$$v_i = \frac{x_i}{Z} \tag{S19c}$$

as in the Tullock contest (Tullock, 1980, Konrad, 2009).

One finds that $d\pi_i/dx_i = b - 0.5d[(n-1)\tilde{x} + 2x] - c_i$. Therefore $D_{0,i} = b - c_i, D_1 = (n-1)d/2, D_2 = d$, and

$$\theta_i = \frac{2(b-c_i)}{d(n+1)}.$$

EGT analysis. Replacing, as before, the term $(n-1)\tilde{x}$ in the payoff function by $X_{i,prev}^{-}$, I find that the best response action and the corresponding Nash equilibria are

$$x_{i,\text{BR}} = \max\left(0, \frac{n+1}{2}\ \theta_i - 0.5X_{i,prev}^{-}\right), \tag{S20a}$$

$$Z_{\text{NE}} = n\ \overline{\theta}, \tag{S20b}$$

$$x_i^* = \theta_i + n(\theta_i - \overline{\theta}). \tag{S20c}$$

Note that for x_i^* to be non-negative, it is required that the minimum $\min(\theta_i) > \frac{n}{n+1}\overline{\theta}$, i.e. variation in θ_i should quickly decrease with n. Once negative values of x_i^* appear, the best response dynamics can become non-equilibrium.

If all individuals have identical coefficients $c_i = c$, then the Nash equilibrium for the total group effort is

$$X_{\text{NE}} = \max\left(0, n\theta\right),$$

while the group effort X_{opt} maximizing the total group payoff is

$$X_{\text{opt}} = \max\left(0, \frac{b-c}{d}\right),$$

that is, $2n/(n+1)$ times smaller.

General analysis. Figure 5 of the main text summarizes the properties of this model. Figure S6 gives examples of the coevolutionary dynamics.

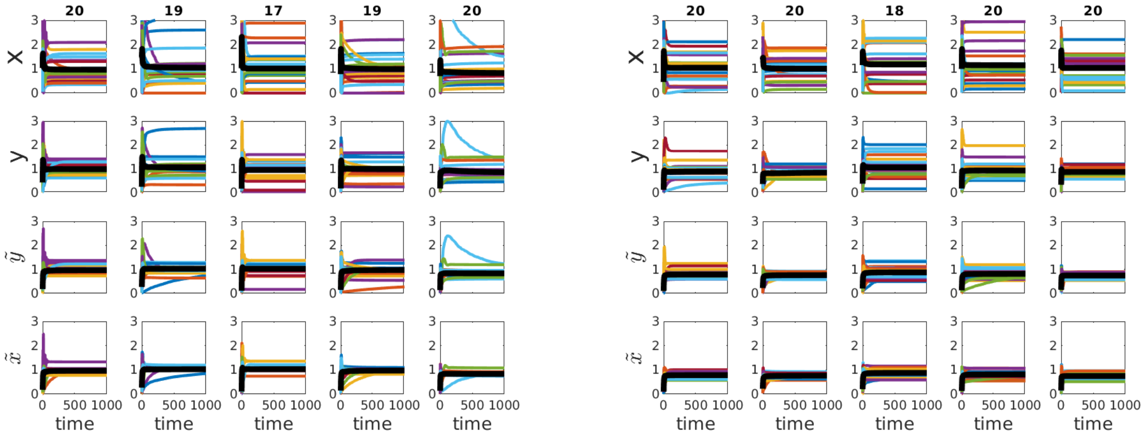

(a) (b)

Figure S6: Examples of coevolutionary dynamics in the Common Pool Resource game corresponding to Figure 5. (a) Five runs with no external influence. (b) Five runs with external influence with $G = 0.5$. Group size $n = 20$, $\varepsilon = 1$. Different colors show different individuals. The thick black lines show the group averages. The numbers on top show the number of contributing individuals at the last time step.

Tragedy of the Commons Game with quadratic costs

In the Tragedy of the Commons games, individuals exploit a resource getting a benefit which increases with individual effort x_i but sharing a cost of its exploitation which increases with the total group effort X (Hardin, 1968).

Assume that individual benefit is linear in individual effort x_i but the cost is quadratic in group effort X:

$$\pi = b_i x_i - 0.5 c_i Z^2.$$

In this model, $d\pi/dx_i = b_i - c_i[(n-1)\tilde{x} + x_i]$ so that $D_0 = b_i, D_1 = c_i(n-1), D_2 = c_i$ and

$$\theta_i = b_i/(c_i n).$$

EGT analysis. Here the best response action is $x_{\text{BR},i} = \max(0, n\theta_i - Z^-_{i,prev})$. In the symmetric version of this model when all θ_i values are the same, the Nash equilibrium for the total group effort is

$$Z_{\text{NE}} = n\theta = b/c.$$

The total group effort maximizing the total group payoff is

$$Z_{\text{opt}} = b/(cn),$$

that is, n times smaller. Individual contributions can take any values as long as they sum up to $Z_{\text{NE},sym}$.

In the asymmetric case, when benefit-to-cost ratios b_i/c_i are different, only an individual with the largest value of θ_i will make an effort θ_i so that the group effort is

$$Z^* = \max(\theta_i).$$

General analysis. Figure S7 summarizes the properties of this model.

Figure S8 show sample trajectories corresponding to Figure S7 with $\varepsilon = 1$.

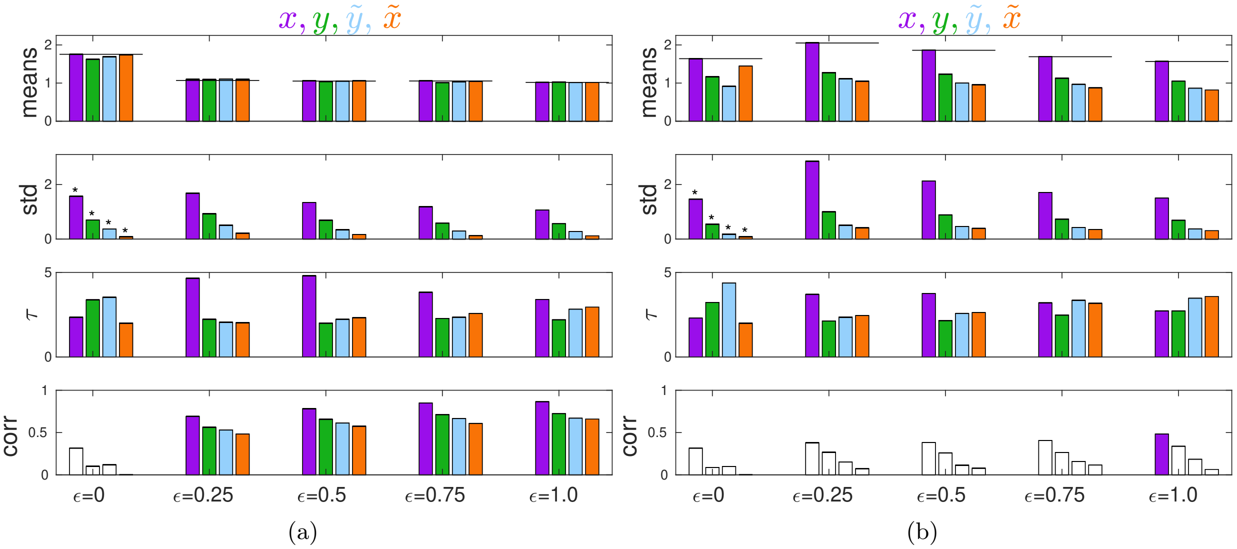

Figure S7: Properties of equilibria in the Tragedy of the Commons game with quadratic costs. (a) No external influence. (b) With external influence promoting decreased effort ($G = 1/n$). From top to bottom: equilibrium means, standard deviations, half-time of convergence to an equilibrium τ, and Kendall correlation with θ for $x, y, \tilde{y}$ and $\tilde{x}$, respectively. Parameter ε measures the importance of each of the normative factors relative to material payoffs. Parameters: $n = 20$, b_i are drawn from a lognormal distribution with mean 1 and variance 0.1, $c_i = 1/n$. The stars on top of the bars for $\varepsilon = 0$ mean that the actual values of standard deviations are 5 times larger than shown. Statistics are calculated over 40 independent runs. Note the difference in convergence time-scales between a and b.

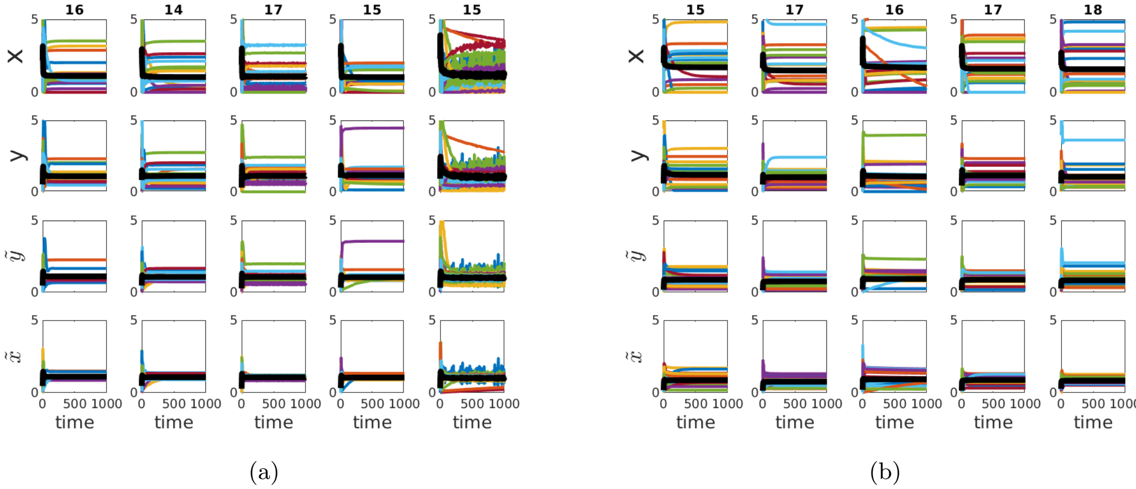

Figure S8: Examples of coevolutionary dynamics in the Tragedy of the Commons game with quadratic costs corresponding to Figure S7. (a) Five runs with no external influence. (b) Five runs with external influence promoting decreased effort at $G = 1/n$. Group size $n = 20$, $\varepsilon = 1$. Different colors show different individuals. The thick black lines show the group averages. The numbers on top show the number of contributing individuals at the last time step.

Tragedy of the Commons game with diminishing returns

Alternatively assume that individual benefit shows a diminishing return while the cost term is linear in group effort Z. Then the payoff function is

$$\pi = (b_i x_i - 0.5 d_i x_i^2) - c_i Z,$$

where b_i, d_i and c_i are individual benefit and cost parameters.

In this game, $d\pi/dx_i = b_i - c_i - d_i x_i$. Therefore $D_0 = b_i - c_i, D_1 = 0, D_2 = d_i$ and

$$\theta_i = \frac{b_i - c_i}{d_i}.$$

EGT analysis. Here the best response action is

$$x_{\text{BR},i} = \max(0, \theta_i),$$

which is also the Nash equilibrium.

In the symmetric version of this game when all coefficients are the same (i.e. $c_i = c, b_i = b, d_i = d$, the Nash equilibrium for the individuals effort is

$$x_{\text{NE},sym} = \frac{b - c}{d}.$$

The individual effort maximizing the total group payoff is

$$x_{\text{opt}} = \frac{b - cn}{d}.$$

General analysis. Figure S9 summarizes the behavior of this model. With no external authority, parameter ε has not effect on average behavior. In contrast to the previous case, individuals respond to the authority and decrease their efforts. The larger ε, the bigger the response. Figure S10 show sample trajectories of the general model corresponding to Figure S9 with $\varepsilon = 1$.

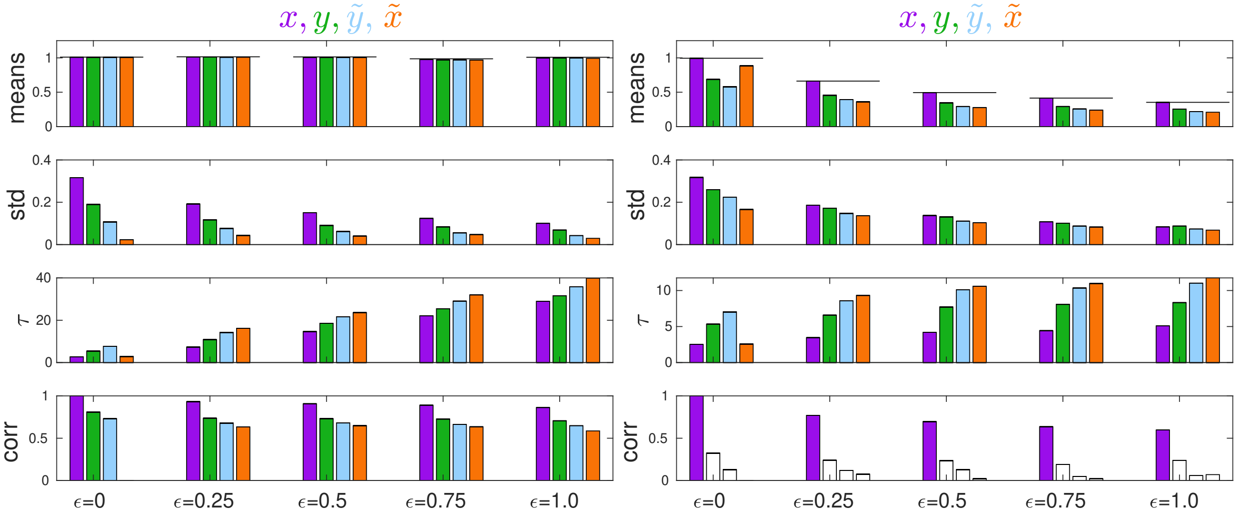

(a) (b)

Figure S9: Properties of equilibria in the Tragedy of the Commons game with diminishing returns. (a) No external influence. (b) With external influence promoting decreased effort at $G = 1/n$. From top to bottom: equilibrium means, standard deviations, half-time of convergence to an equilibrium τ, and Kendall correlation with θ for $x, y, \tilde{y}$ and $\tilde{x}$, respectively. The thin black horizontal lines show the theoretical predictions for x. Parameter ε measures the importance of each of the normative factors relative to material payoffs. Parameters: group size $n = 20$, parameters b_i are drawn from a lognormal distribution with mean $n + 1$ and standard deviation $0.1 \times \sqrt{n+1}$, $c_i = 1, d_i = n$.

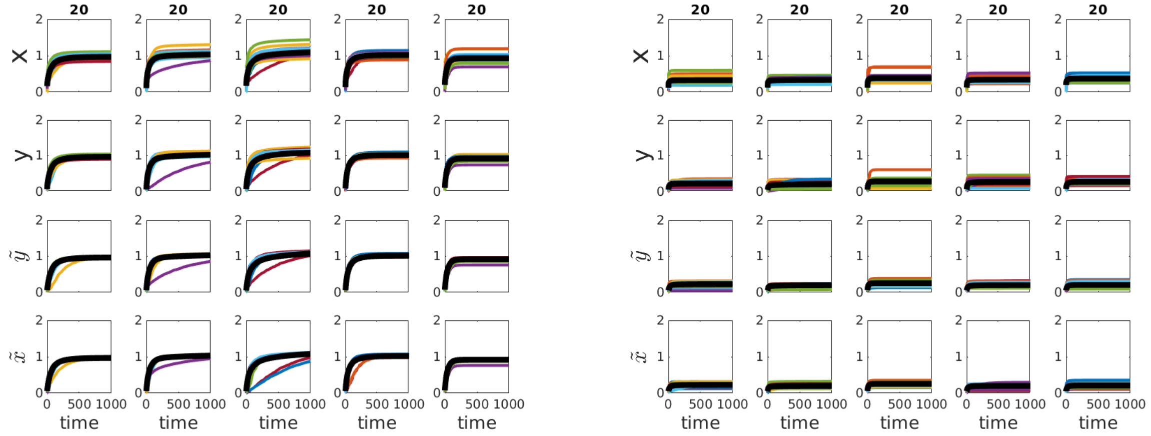

(a) (b)

Figure S10: Examples of coevolutionary dynamics in the Tragedy of the Commons game with diminishing returns. (a) No external influence. (b) Five runs with external influence promoting decreased effort at $G = 1/n$. Group size $n = 20$, $\varepsilon = 1$. Different colors show different individuals. The thick black lines show the group averages. The numbers on top show the number of contributing individuals at the last time step.

Trade-offs between public and private production

In this game (Willinger and Ziegelmeyer, 1999, 2001, Laury and Holt, 2008, McGinty and Milam, 2013) the payoff function is a sum of two components coming from public and private production efforts:

$$\pi_i(x_i) = v_i \underbrace{(BZ - 0.5DZ^2)}_{\text{collective production}} + \underbrace{b_i y_i - 0.5 d_i y_i^2}_{\text{private payoff}},$$

where $Z = \sum x_j$, x_i is the contribution to collective production and v_i is the share/valuation of this production for individuals i . The effort not invested in public production, $y_i = R_i - x_i$, where R_i is a constant endowment of individual i, is invested in private production; b_i and d_i are the corresponding benefit and cost coefficients.

Following McGinty and Milam (2013), assume egalitarian division of pubic goods (i.e. $v_i = 1/n$) and that b_i/d_i is a constant.

Then

$$\frac{d\pi_i}{dx_i} = \underbrace{v_i B - b_i + d_i R_i}_{D_0} - \underbrace{v_i D(n-1)}_{D_1}\ \tilde{x} - \underbrace{(d_i + v_i D)}_{D_2}\ x.$$

In this model,

$$\theta_i = \frac{v_i B - b_i + d_i R_i}{d_i + v_i D n} = \frac{R_i + \frac{v_i B - b_i}{d_i}}{1 + n\frac{v_i D}{d_i}} \equiv \frac{\lambda_i}{1 + n\kappa_i}.$$

with the obvious meaning of λ_i and κ_i.

EGT analysis. The best response action for individual i is

$$x_{\text{BR},i} = \frac{\lambda_i - \kappa_i X_i^-}{1 + \kappa_i}. \tag{S21a}$$

From its meaning, x must stay between 0 and R. Generalizing McGinty and Milam (2013) approach under the assumption that all $x_{\text{BR},i} > 0$, I find that the total group effort at the Nash equilibrium can be written as

$$Z_{\text{NE}} = \frac{\sum \lambda_i}{1 + \sum \kappa_i}. \tag{S21b}$$

while the individual effort is

$$x_{i,\text{NE}} = \lambda_i - \kappa_i Z_{\text{NE}}. \tag{S21c}$$

General analysis. Figure S11 summarizes the properties of this model. Figure S12 shows sample trajectories of the general model corresponding to Figure S11 with $\varepsilon = 1$.

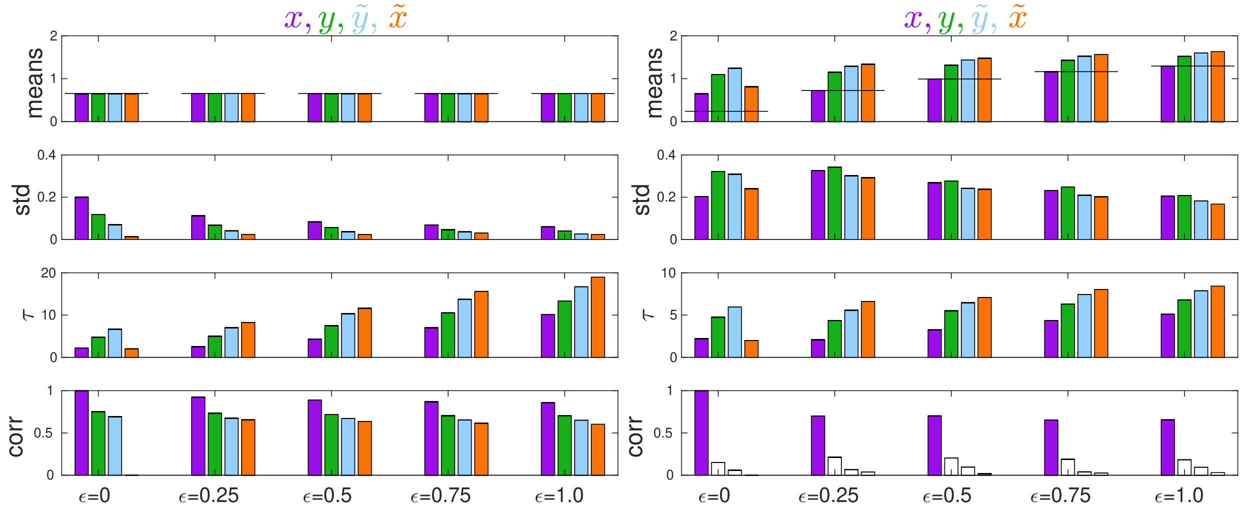

(a) (b)

Figure S11: Properties of equilibria in the Trade-offs game. (a) No external influence. (b) With external influence promoting effort $G = 2$. From top to bottom: equilibrium means, standard deviations, half-time of convergence to an equilibrium τ, and Kendall correlation with θ for $x, y, \tilde{y}$ and $\tilde{x}$, respectively. The thin black horizontal lines show the theoretical predictions for x. Parameter ε measures the importance of each of the normative factors relative to material payoffs. Parameters: $n = 50$, b_i are drawn from a lognormal distribution with mean 1 and variance 0.1, $B_m = 1, D = 1, d = 1, R = 2$ while parameters v_i are drawn from a broken stick distribution on $[0, 1]$.

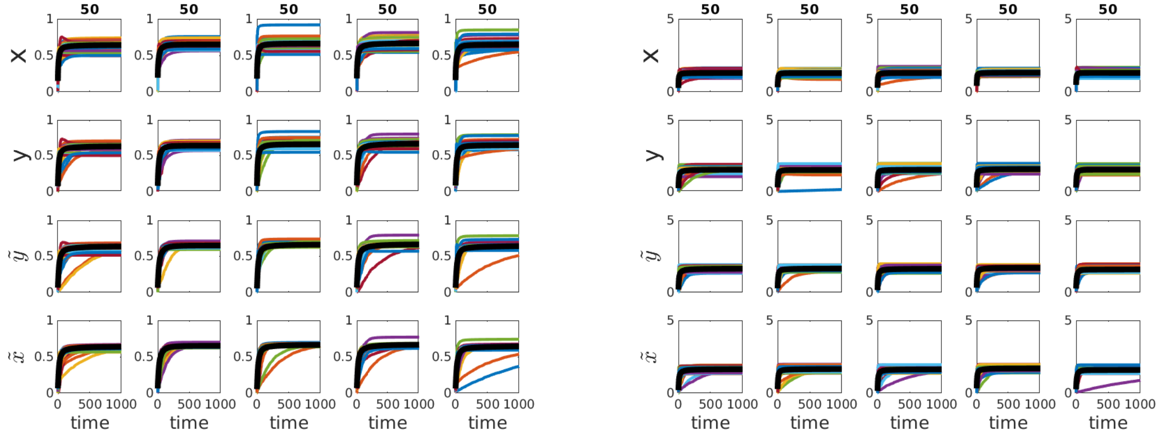

(a) (b)

Figure S12: Examples of coevolutionary dynamics in the Trade-offs Game corresponding to Figure S11. (a) No external influence. (b) Five runs with external influence promoting decreased contribution to private production at $G = 1/n$. Group size $n = 50, \varepsilon = 1$. Different colors show different individuals. The thick black lines show the group averages. The numbers on top show the number of contributing individuals at the last time step.

Games with linear payoff functions: Dictator, Take-or-Give, Rule Obedience, and Public Goods

Linear payoff functions emerge in a number of simple games commonly used in experimental economics research. Some examples are given next.

Dictator game. Here an individual with an endowment R decides on how much to give to another individual. If x_i is the donation, then the payoff function is $\pi(x_i, \tilde{x}_i) = R - x_i$, so that $d\pi_i/dx_i = -1$ and $D_{0,i} = -1$.

Take-or-Give game. In this game (Bicchieri *et al.*, 2020), each individual decided on whether to contribute to a pool of money marked to be given to a charity ($x_i > 0$) or take the money from this pool for personal use ($x_i > 0$). One can write the payoff as $\pi(x_i, \tilde{x}_i) = R - x_i$, so that $d\pi_i/dx_i = -1$ and $D_{0,i} = -1$.

Rule Obedience game. In this game designed and studied by Kimbrough and Vostroknutov (2016, 2019) individuals can follow verbal instructions (such as "wait for a crosswalk light to turn green") and earn a certain reward or ignore instructions and get a higher reward. Let x_i be the waiting time. Then the payoff function in this game can be written as $\pi(x_i, \tilde{x}_i) = R - x_i$, so that $d\pi_i/dx_i = -1$ and $D_{0,i} = -1$.

Linear Public Goods game. In this classical game, the payoff function is

$$\pi(x_i, \tilde{x}_i) = v_i bZ - c_i x_i, \tag{S22}$$

where b, v_i and c_i are constant parameters. Then $d\pi_i/dx_i = bv_i - c_i$. A standard assumption in behavioral studies is that $D_{0,i} = bv_i - c_i < 0$.

In all these games, I predict that in the absence of additional forces, contributions x_i and attitudes y_i and beliefs $\tilde{y}_i, \tilde{x}_i$ will evolve to the minimum values, i.e. zero. However in the presence of an external influence, the equilibrium contribution can be positive.

Figure S13a illustrates the properties of this model when $G = 2$. Figure S14 gives examples of corresponding trajectories.

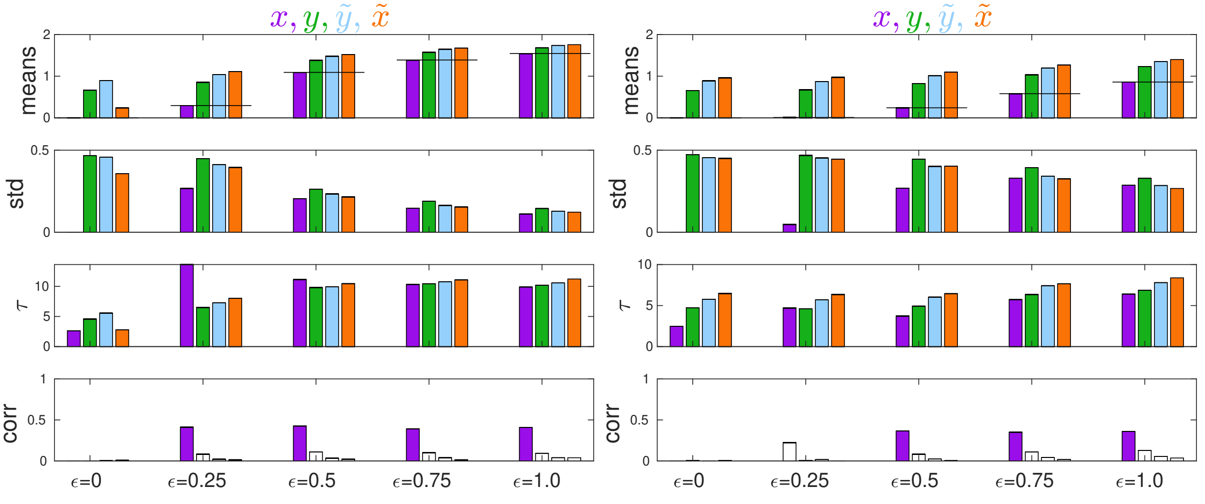

(a) (b)

Figure S13: (a) Linear Public Goods game with external influence with $G = 2$. Parameters: group size $n = 100, D_1 = D_2 = 0$ for all i while D_0 are drawn from a uniform distribution on $[-2, 0]$. (b) Continuous Prisoner's Dilemma game with external influence with $G = 2$. Parameters: group size $n = 50, D_2$ for all i while parameters D_0, D_1 are drawn from lognormal distributions with mean -1 and 1, respectively, and standard deviation 0.1. These expectations arise if the expectations of S, P, R and T and $0, 1, 3$ and 5, respectively. From top to bottom: equilibrium means, standard deviations, correlations with D_0, and the half-time of convergence for $x, y, \tilde{y}$ and $\tilde{x}$, respectively. The thin black horizontal lines show the theoretical predictions for x. Parameter ε measures the importance of each of the normative factors relative to material payoffs. Statistics are calculated over 100 last time steps over 40 independent runs each of length 1,000 time steps.

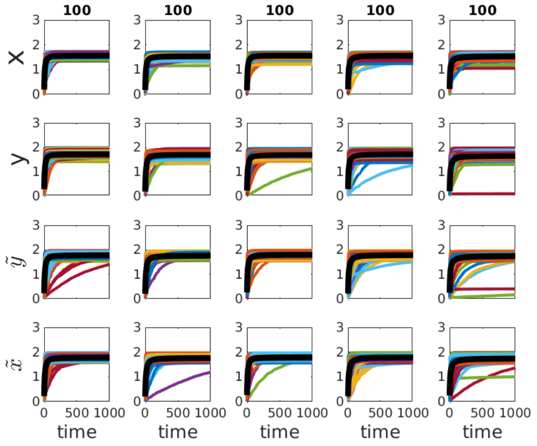

Figure S14: Examples of coevolutionary dynamics in the Linear Public Goods Game with external influence at $G = 2$ corresponding to Figure S13a. Group size $n = 100, \varepsilon = 1$. Different colors show different individuals. The thick black lines show the group averages. The numbers on top show the number of contributing individuals at the last time step. Parameters D_0 are chosen from a uniform distribution on $[-2, 0]$. $D_1 = D_2 = 0$ for all i.

Continuous Prisoner's Dilemma game

Verhoeff (1998) introduced a continuous variant of the Prisoner's Dilemma which he called the Trader's Dilemma. In this game, each of the two players chooses an effort x within a unit interval $[0, 1]$. The payoff to the player A who makes effort x_A against player B who makes effort x_B can be written as

$$\pi(x_A, x_B) = x_A x_B R + x_A(1 - x_B)S + (1 - x_A)x_B T + (1 - x_A)(1 - x_B)P.$$

Parameters R, S, T, P correspond to the reward, sucker's pay, temptation and punishment payoffs in the standard Prisoner's Dilemma (with $T > R > P > S$). One interpretation of this game is that the players are trade partners. One of them bring a box of rice, the other a box of beans. An action consists of exchanging boxes filled with a certain amount of merchandise. Complete cooperation corresponds to bringing a box completely filled with the promised merchandise. Complete defection corresponds to bringing an empty box.

Adopting this model to my framework, player i will expect a payoff which can be written as

$$\pi(x_i, \tilde{x}_i) = x_i \tilde{x}_i R_i + x_i(1 - \tilde{x}_i)S_i + (1 - x_i)\tilde{x}_i T_i + (1 - x_i)(1 - \tilde{x}_i)P_i.$$

where I allow for heterogeneity in players payoffs. In this model,

$$D_0 = S_i - P_i < 0, D_1 = T_i - R_i - P_i + S_i, D_2 = 0.$$

In this game, $D_0 - D_1\tilde{x}_i < 0$ for all $\tilde{x}_i$. Therefore the players will evolve to a state with zero efforts in the standard game theoretic approach. The same outcome is predicted in my model if there is no external influence. [Note that in games of partial cooperation studied by Stark (2010), $D_1 > 0$. In these games, defection dominates cooperation, but an intermediate fraction of cooperators would maximize the group payoff.]

Figure S13b of the main text illustrates the properties of this model with external influence with $G = 2$. Figure S15 gives examples of corresponding trajectories.

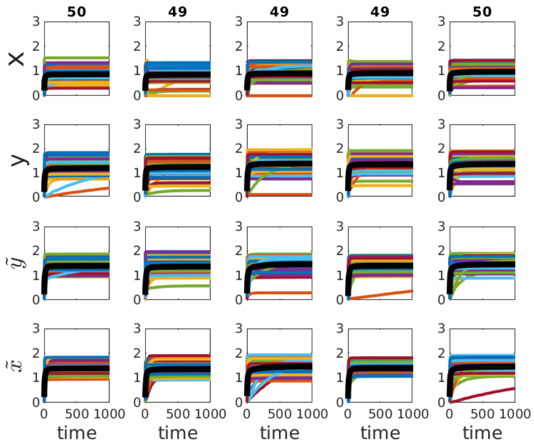

Figure S15: Examples of coevolutionary dynamics in the continuous Prisoner's Dilemma game with external influence at $G = 2$ corresponding to Figure S13b. Group size $n = 50, \varepsilon = 1$. Different colors show different individuals. The thick black lines show the group averages. The numbers on top show the number of contributing individuals at the last time step.

“Us v. nature” game

This game (Gavrilets, 2015, Gavrilets and Richerson, 2017) is similar to the linear public goods game, except that the production function saturates at a constant level as the group efforts increase:

$$P = \frac{Z}{Z + Z_0},$$

where Z_0 is a constant half-effort parameter (at $Z = Z_0$, the group produces half of the maximum amount of resource). Because of the non-linearity of this game, my analytical results do not apply and there is no analogue of parameter θ here.

EGT analysis. The best response effort in this game is

$$x_i = \max\left[0, Z_0\left(\sqrt{R_i} - 1\right) - Z^-_{i,prev}\right],$$

where $R_i = b_i/(c_i Z_0)$ is the ratio of the individual benefit and the group's cost at half-effort.

In the symmetric case, the group effort at the Nash equilibrium is

$$Z^*_{sym} = Z_0(\sqrt{R} - 1).$$

In the asymmetric case, only the individual with the largest value of R_i will make an effort while all other individuals will free-ride:

$$Z^*_{asym} = Z_0(\sqrt{\max{(R_i)}} - 1).$$

General case. With additional normative forces, finding the normalized best response effort requires one to solve the cubic:

$$bZ_0 - (c - S_1 + S_2 x)(Z_0 + x + X^-)^2 = 0,$$

where $S_1 = A_1 y + A_2 \tilde{y} + A_3 \tilde{x} + A_4 G, S_2 = \sum_{j=1}^4 A_j$. This can be done numerically. Note that all coefficients here except for Z_0 are individual-specific.

Figure S16 illustrates the properties of this model. Figure S17 gives examples of corresponding trajectories.

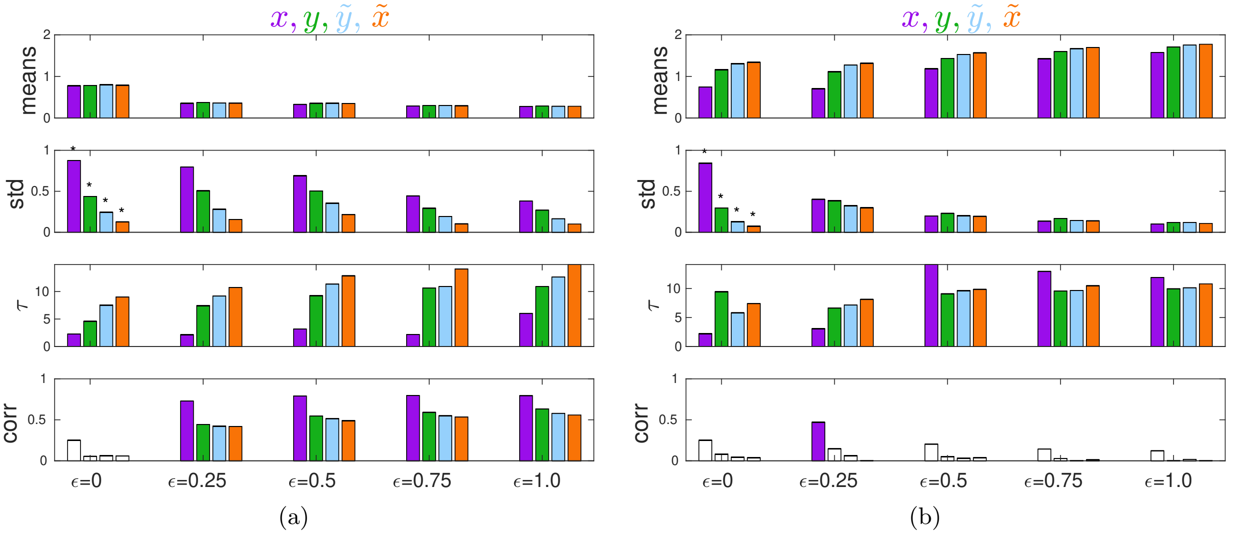

Figure S16: Properties of equilibria in the "Us vs. nature" game. (a) No external influence. (b) With external influence ($G = 2$). From top to bottom: mean, standard deviation, half-time of convergence to an equilibrium τ, and correlation with θ for x (purple), y (green), $\tilde{y}$ (blue) and $\tilde{x}$ (orange), respectively. Parameter ε measures the importance of each of the normative factors relative to material payoffs in the utility function. Bars with no color mean the corresponding correlations are statistically insignificant (at 0.05). Group size $n = 32, b = 32n, c = 1, X_0 = n/4$. Parameters v_i are jointly drawn from a broken stick distribution on $[0, 1]$. Statistics are calculated over 40 independent runs. The stars on top of the bars for $\varepsilon = 0$ mean that the actual values of standard deviations are 5 times larger than shown.

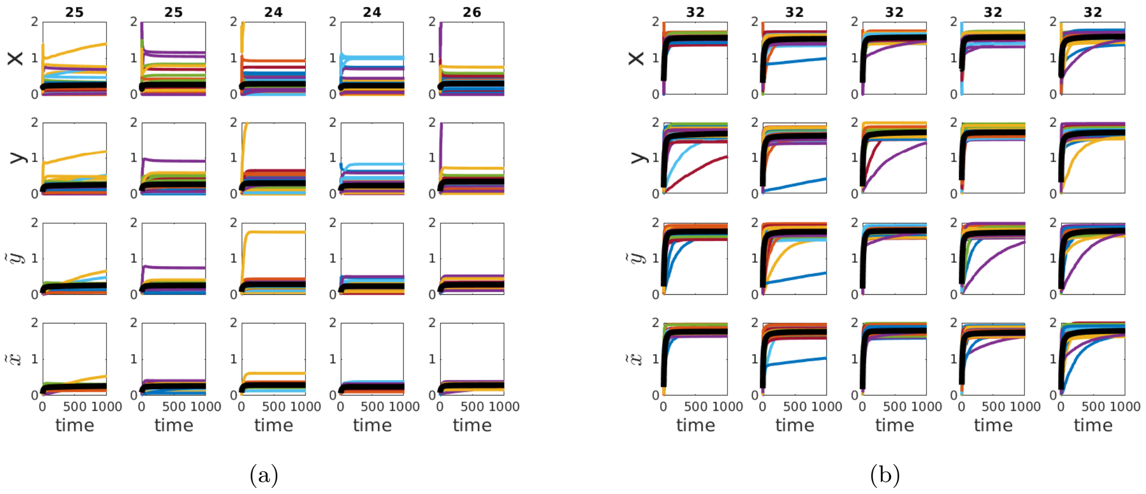

Figure S17: Examples of coevolutionary dynamics in the "us vs. nature" game. (a) No external influence. (b) Five runs with external influence with $G = 2$. $n = 32, \varepsilon = 1$. The numbers on top show the number of contributing individuals (with $s > 0$) at the last time step.

References

Anderson, S. P., Goeree, J. K., and Hol, C. A. (1998). A theoretical analysis of altruism and decision error in public goods games. *Journal of Public Economics*, **70**, 297–323.

Andreoni, J., Nikiforakis, N., and Siegenthaler, S. (2021). Predicting social tipping and norm change in controlled experiments. *Proceedings of the National Academy of Sciences USA*, **118**, e2014893118.

Apesteguia, J. and Maier-Rigaud, F. P. (2006). The tole of rivalry: Public goods versus common-pool resources. *Journal of Conflict Resolution*, **50**, 646–663.

Bicchieri, C., Dimant, E., Gachter, S., and Nosenzo, D. (2020). Observability, social proximity, and the erosion of norm compliance. *https://dx.doi.org/10.2139/ssrn.3355028.*

Calabuig, V., Olcina, G., and Panebianco, F. (2018). Culture and team production. *Journal of Economic Behavior and Organization*, **149**, 32–45.

Esteban, J. and Ray, D. (2001). Collective action and the group size paradox. *American Political Science Review*, **95**, 663–672.

Gavrilets, S. (2015). Collective action problem in heterogeneous groups. *Proceedings of the Royal Society London B*, **370**, 20150016.

Gavrilets, S. and Fortunato, L. (2014). A solution to the collective action problem in between-group conflict with within-group inequality. *Nature Communications*, **5**, article 3526 (doi:10.1038/ncomms4526).

Gavrilets, S. and Richerson, P. J. (2017). Collective action and the evolution of social norm internalization. *Proceedings of the National Academy of Sciences USA*, **114**, 6068–6073.

Hardin, G. (1968). Tragedy of commons. *Science*, **162**(3859), 1243–1248.

Kimbrough, E. O. and Vostroknutov, A. (2016). Norms make preferences social. *Journal of the European Economic Association*, **14**, 608–638.

Kimbrough, E. O. and Vostroknutov, A. (2019). A theory of injunctive norms.

Konrad, K. (2009). *Strategy and Dynamics in Contests*. Oxford University Press, Oxford.

Kuran, T. and Sandholm, W. H. (2008). Cultural integration and its discontents. *Review of Economic Studies*, **75**(1), 201–228.

Laury, S. K. and Holt, C. A. (2008). Chapter 84. Voluntary provision of public goods: Experimental results with interior nash equilibria. volume 1 of *Handbook of Experimental Economics Results*, pages 792 – 801. Elsevier.

MacArthur, R. (1957). On the relative abundance of bird species. *Proceedings of the National Academy of Sciences USA*, **43**, 293–295.

McGinty, M. and Milam, G. (2013). Public goods provision by asymmetric agents: experimental evidence. *Soc Choice Welf*, **40**, 1159–1177.

Stark, H.-U. (2010). Dilemmas of partial cooperation. *Evolution*, **64**, 2458–2465.

Tullock, G. (1980). Efficient rent seeking. In J. M. Buchanan, R. D. Tollison, and G. Tullock, editors, *Toward a theory of the rent-seeking society*, pages 97–112. Texas A & M University, College Station.

Verhoeff, T. (1998). The trader's dilemma: A continuous version of the prisoner's dilemma. Technical report, Faculty of Mathematics and Computing Science, Technische Universiteit Eindhoven, The Netherlands.

Walker, J. M., Gardner, R., and Ostrom, E. (1990). Rent dissipation in a limited-access common-pool resource: Experimental evidence. *Journal of Environmental Economics and Management*, **19**, 203–211.

Willinger, M. and Ziegelmeyer, A. (1999). Framing and cooperation in public good games: an experiment with an interior solution. *Economics Letters*, **65**, 323–328.

Willinger, M. and Ziegelmeyer, A. (2001). Association strength of the social dilemma in a public goods experiment: An exploration of the error hypothesis. *Experimental Economics*, **4**, 131–144.
